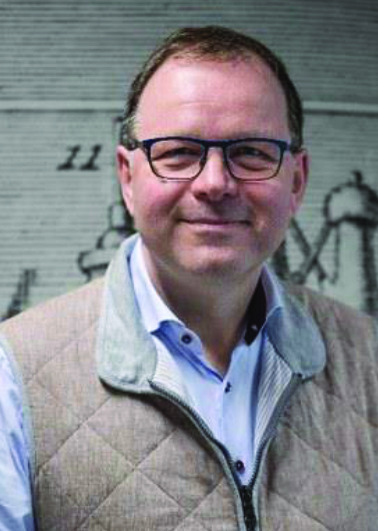# Carbon Dioxide as a Sustainable Reagent in Circular Hydrometallurgy

**DOI:** 10.1002/cssc.202400931

**Published:** 2024-11-07

**Authors:** Rodolfo Marin Rivera, Koen Binnemans

**Affiliations:** ^1^ Department of Chemistry KU Leuven Celestijnenlaan 200F B-3001 Heverlee Belgium

**Keywords:** Carbon dioxide, Circular hydrometallurgy, Greenhouse gas emissions, Zero-waste valorisation

## Abstract

This review highlights the use of CO_2_ as a reagent in hydrometallurgy, with emphasis on the new concept of circular hydrometallurgy. It is shown how waste CO_2_ can be utilised in hydrometallurgical operations for pH control or regeneration of acids for leaching. Metal‐rich raffinate solutions generated after removal of the valuable metals can serve as feedstocks for mineral carbonation, providing alternative avenues for CO_2_ sequestration. Furthermore, CO_2_ can also be used as a renewable feedstock for the production of chemical reagents that can find applications in hydrometallurgy as lixiviant, as precipitation reagent or for pH control. Mineral carbonation can be combined with chemical reactions involving metal complexation reagents, as well as with solvent extraction processes for the concurrent precipitation of metal carbonates and acid regeneration. An outlook for future research in the area is also presented.

## Graphical abstract

Carbon dioxide is a useful reagent in hydrometallurgy and it can help to transform linear hydrometallurgical flowsheets into circular ones. Examples of applications of CO_2_ are pH control, the regeneration of acids used as lixiviants, or as a reagent for precipitation of metal carbonates. A new development is the solvent‐extraction‐assisted formation of metal carbonates.

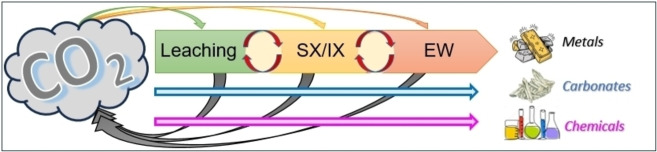

## 1 Introduction

Metals are extracted from ores, concentrates, industrial process residues by pyrometallurgical (high‐temperature) and/or hydrometallurgical (water‐based) processes, whereas refining of metals is mostly carried out with hydrometallurgical methods (solvent extraction, ion exchange, electrowinning), particularly if highly pure metals are required.^[1]^ Many hydrometallurgical processes are consuming large amount of acids or bases, which entails the generation of waste that can have a hazardous impact on the environment if not properly handled. The hydrometallurgical processes used for extraction and refining of metals are often energy intensive, especially the electrowinning of metals from solution. If the energy requires for the processes is generated by burning fossil fuels (oil, coal and natural gas), large amounts of carbon dioxide (CO_2_) are generated. It is well known that CO_2_ is a greenhouse gas (GHG), and anthropogenic CO_2_ emission strongly contributes to global warming. Hydrometallurgical processes can have a large carbon footprint not only because of the energy consumption for their production, but also because of the production of the chemical reagents needed for the extraction, separation and purification of these metals. Examples are lime (either in the form of CaO or Ca(OH)_2_) and MgO that are widely used as neutralisation reagents and/or for pH control,[^2^, ^3^] but these are produced from carbonate minerals that require high‐temperature calcination processes. The CO_2_‐footprint for lime production can be as high as 1900 kg CO_2_‐eq per tonne of lime produced.^[4]^ Also, most commercial diluents used in solvent extraction are petroleum‐derived products, which require several processing steps for refining and can entail the generation of more than 460 kg CO_2_‐eq per tonne of diluent produced.^[5]^

**Figure 1:**
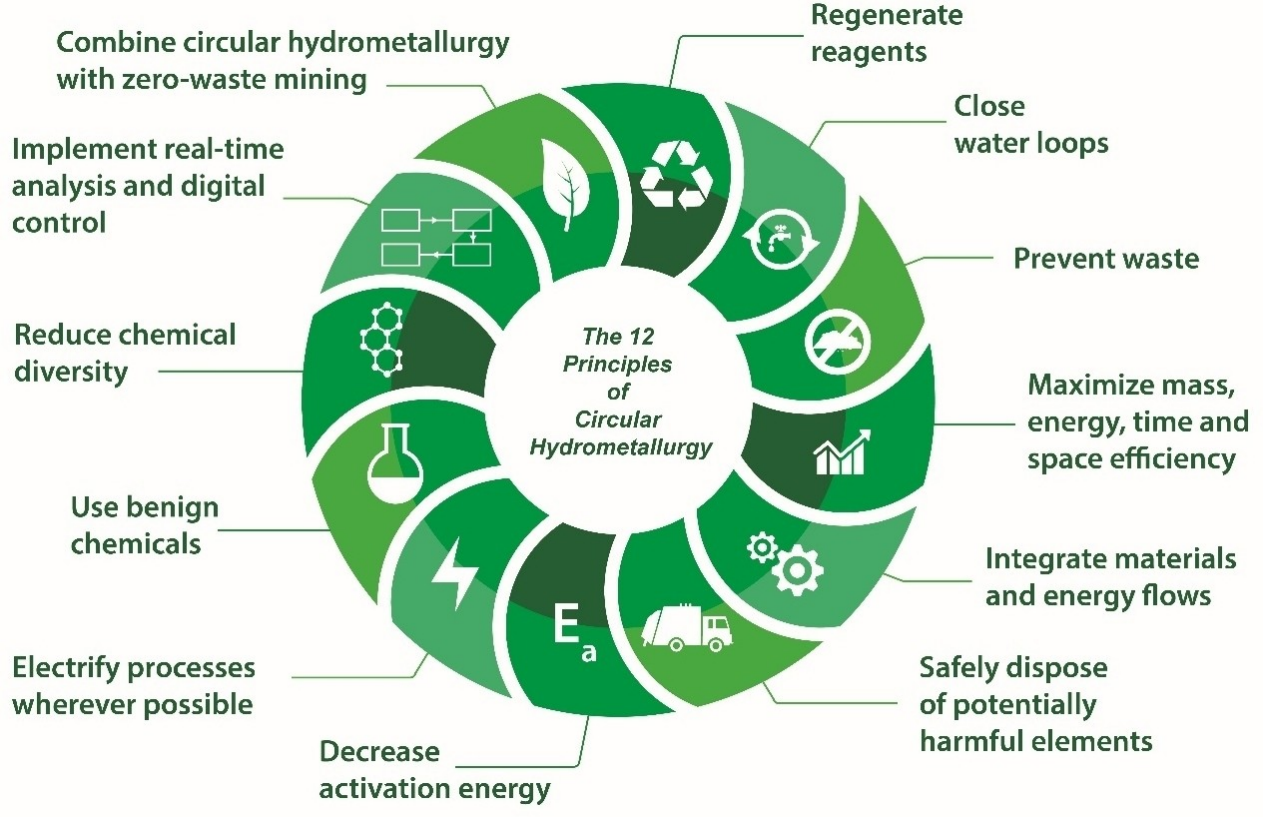
The 12 principles of circular metallurgy. Reproduced from reference [6].

To make the extraction and refining of metals more sustainable, the concept of “circular hydrometallurgy” was recently introduced.^[6]^ Circular hydrometallurgy is defined as the design of process flowsheets that are both energy and resource‐efficient, consuming minimal amounts of reagents and generating minimal waste. As a guideline for designed circular hydrometallurgical processes, 12 principles have been defined (see Figure 1): (1) regenerate reagents, (2) close water loops, (3) prevent waste, (4) maximize mass, energy, space, and time efficiency, (5) integrate materials and energy flows, (6) safely dispose of potentially harmful elements, (7) decrease activation energy, (8) electrify processes wherever possible, (9) use benign chemicals, (10) reduce chemical diversity, (11) implement real‐time analysis and digital process control, and (12) combine circular hydrometallurgy with zero‐waste mining. By applying the principles of circular hydrometallurgy, it becomes evident that CO_2_ is not just a waste product and greenhouse gas that needs to be avoided at all means, but that CO_2_ can also be a valuable reagent on its own and that hydrometallurgical processes can help to reduce CO_2_ emissions via mineral carbonation.[^7^, ^8^]

Mineral carbonation has the potential to capture and safely store large volumes of CO_2_. It is based on the principles of natural rock weathering, where the CO_2_ dissolved in rainwater reacts with alkaline rocks to form carbonate minerals.[^9^, ^10^] The active alkaline elements, calcium and magnesium, are the fundamental reactants for the mineral carbonation reaction. Although the reaction is thermodynamically favoured, its kinetics are slow and on the geological time scale.^[11]^ Researchers have offset this limitation by accelerating the carbonation reaction with minimal energy and feedstock consumption.[^7^, ^12^] Naturally occurring silicate minerals rich in calcium and magnesium, such as olivine, serpentine and wollastonite, are generally selected for carbonation due to their abundance in nature.[^8^, ^13^, ^14^] Industrial waste residues, such as mine tailings, steelmaking slags, fly ash, gypsum and bauxite residues, have also emerged as an alternative source due to their relatively high concentration of alkali and alkaline earth metals, and their availability near CO_2_ producers.[^15^, ^16^] It must be noticed, however, that the concentration of CO_2_ in flue gases is low and comprises often only a few percent (around 12‐14 % in flue gas from coal‐fired power plants), which implies that large amounts of emissions have to be treated to separate the CO_2_ formed during combustion. This implies that the whole sequestration process may result in more CO_2_ emissions than the sequestered CO_2_ due to the energy requirement for separating CO_2_ from the other gases, so that there is no net benefit in terms of CO_2_ emission control.

This review gives an overview of the application of CO_2_ in hydrometallurgical processes for the extraction and refining of metals. It is described how CO_2_ can be used for removal of metals from raffinates of hydrometallurgical processes by mineral carbonation reactions, for neutralisation of alkaline solutions and solids, for regeneration of acids, as part of lixiviants, and other applications that aid to make hydrometallurgical processes circular.

## 2 CO_2_ as Component of Chemical Reagents

### 2.1 Carbonic Acid

Carbonic acid (H_2_CO_3_) is formed during the dissolution of CO_2_ in water, according to Equations (1) and (2). The fraction of CO_2_ that reacts with water to form carbonic acid depends on factors such as temperature, pressure, and the presence of other dissolved substances. At ambient conditions (1 atm and 20–25 °C), the solubility of CO_2_ in water increases as the temperature decreases. The equilibrium constant for the reaction between CO_2_ and water (*K*_eq_) is 1.7×10^−3^ mol L^−1^, meaning that the vast majority of dissolved CO_2_ remains in the form of neutral CO_2_ molecules (CO_2(aq)_), with only a small fraction reacting with water to form carbonic acid. Carbonic acid has a low stability as it decomposes into H_2_CO_3_/HCO_3_^−^ with p*K*_a1_ = 6.35 (Equation (3)) and HCO_3_^−^/CO_3_^2−^ at p*K*_a2_ = 10.2 (Equation (4)), at 25 °C.

(1) $$CO_{2\left(g\right)}\vec{\leftarrow }CO_{2\left(aq\right)}$$

(2) $$CO_{2\left(aq\right)}+H_{2}O\vec{\leftarrow }H_{2}CO_{3\left(aq\right)}$$

(3) $$H_{2}CO_{3\left(aq\right)}\vec{\leftarrow }HCO_{3\left(aq\right)}^{-}+H_{\left(aq\right)}^{+}$$

(4) $$HCO_{3\left(aq\right)}^{-}\vec{\leftarrow }CO_{3\left(aq\right)}^{2-}+H_{\left(aq\right)}^{+}$$

**Figure 2:**
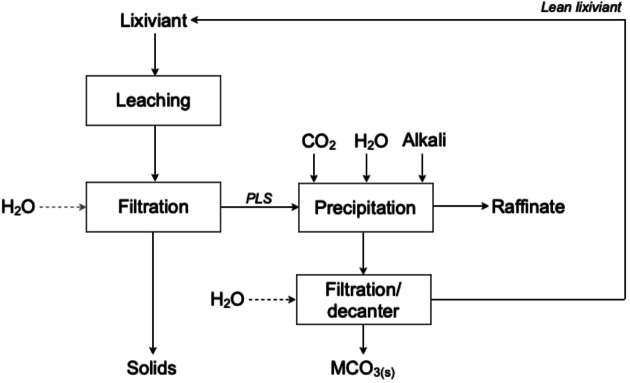
Typical process flowsheet for indirect mineral carbonation (adapted from references [17,31]) with permission from Elsevier, copyright 2017, and the Royal Society of Chemistry).

Most of the research on the utilisation of aqueous CO_2_ and, therefore, on H_2_CO_3_, has focused on mineral carbonation. Due to its mild acidity, H_2_CO_3_ complies with the principle Use Benign Chemicals in circular hydrometallurgy. *In situ* mineral carbonation involves injecting CO_2_ directly into geological formations containing reactive minerals. The CO_2_ reacts with the minerals in the subsurface, forming carbonate minerals. This process can be carried out in deep geological formations, such as depleted oil and gas reservoirs, saline aquifers, or basalt formations. The methodology offers potential advantages in terms of storage security and capacity, but requires careful site selection and characterisation.[^7^, ^17^] *Ex situ*, or indirect mineral carbonation, on the other hand, has received widespread attention due to the relatively mild reaction conditions, high carbonation yields and formation of high‐quality products. The method comprises two successive steps, as described in Figure 2: (1) dissolution of metals (mostly calcium and magnesium) in (mild) acidic conditions and; (2) carbonation with aqueous CO_2_ in a basic or weakly basic environment.^[18]^ Different solvents have been studied as lixiviants for the extraction of metals, such as mineral acids commonly used in mineral processing (*e.g*., HCl, H_2_SO_4_, HNO_3_), weak acids (*e.g*., HCOOH, CH_3_COOH) and bases (*e.g*., NaOH, KOH).[^19^, ^20^, ^21^, ^22^, ^23^, ^24^, ^25^, ^26^, ^27^] The formation of carbonates takes place in the subsequent step, through the introduction of CO_2_ into the aqueous solution normally at relatively high pH values when carbonic acid starts decomposing into hydrogen carbonate ions. In the absence of pH control, the formation of carbonates is unfavourable and/or low‐purity carbonates are obtained. Hence, it is common practice to add alkaline reagents for pH control during carbonation. Chen *et al*. reported the precipitation of MnCO_3_ from electrolyte manganese residue treated by CO_2_ in the presence of NaOH and CaO.^[28]^ Rivera *et al*. described the addition of NaOH for enhancing the carbonation of calcium in a HCl‐based aqueous solution, as well as the addition of organic amine solvents, such as ethanolamine and aminoethylethanolamine.^[29]^ The increase in the solution′s pH by addition of alkaline reagents improves carbonation yields, while producing carbonate compounds with different morphologies. Depending on the leaching conditions, other elements can be solubilised as well, which end‐up either in the wastewater and/or are incorporated in the lattice structure of carbonate products.^[30]^

#### 2.1.1 Metal Extraction from Silicate Materials

There are only few studies on the utilisation of carbonic acid as a leaching reagent (lixiviant) for the extraction of metals other than calcium and magnesium. Walder and Bialecki patented a method for the dissolution of iron and magnesium from silicate minerals by integrating the introduction of CO_2_ with high voltage pulses, which allowed the concurrent dissolution of the silicate matrix, and the release of Fe(II) and Mg(II) ions into the solution in less than 30 min.^[32]^ Iron could be recovered from the solution as iron(III) hydroxide, Fe(OH)_3_, by introducing oxygen gas in the system, while magnesium could be extracted by solvent extraction and/or precipitation. Due to the integration of CO_2_ with high voltage pulses, the activation energy of the reaction is significantly reduced leading to an enhancement of the reaction rate. Hence, the process complies with the principles Use Benign Chemicals, Decrease Activation Energy and Maximise Time Efficiency.

The dissolution of silicon and aluminium from montmorillonite mineral in H_2_CO_3_ was studied by Ni *et al*,^[33]^ who reported that at a given H_2_CO_3_ concentration, silicon dissolution increases when increasing the temperature from 25 to 45 °C, while aluminium remained unreactive under these conditions. Huijgen *et al*. studied the leaching of products and reaction mechanism of metals during the carbonation of steel slag.^[34]^ Carbonation was shown to reduce the leaching of most alkaline earth metals (with exception of magnesium) by conversion of calcium‐rich phases into calcite. It was also observed that leaching of vanadium from the slag increased substantially upon carbonation, most likely due to the dissolution of a calcium vanadate mineral. Santos *et al*. reported the utilisation of mineral carbonation at high CO_2_ partial pressure (200 bar) and temperature (200 °C) as a pretreatment for the extraction of nickel from a low‐grade silicate ore.^[35]^ The carbonation efficiency of the olivine mineral was enhanced by introducing 1 mol L^−1^ NaCl under autoclaving conditions.

#### 2.1.2 Treatment of Bauxite Residue

Bauxite residue (red mud) is a highly alkaline residue obtained after extraction of alumina from bauxite ore in the Bayer process.^[36]^ A small number of studies has focused on the use of carbonic acid as neutralisation reagent for the safe disposal of bauxite residue,[^37^, ^38^, ^39^] i. e. principles Safely Dispose of Potentially Harmful Elements and Prevent Waste. From the perspective of metal recovery, the alkalinity of bauxite residue (pH between 10 and 14) represents an important issue in acidic leaching, because part of the acid added must be used for neutralisation of the alkaline products left behind after the Bayer process. This leads to a large acid consumption and makes the recovery of metals from bauxite residue economically not feasible. Rivera *et al*. studied the use of carbonic acid as a neutralisation reagent for bauxite residue before acidic leaching for the recovery of rare‐earth elements (REEs).^[40]^ The pH of the bauxite residue was reduced from about 11.0 to. 9.0 by reaction of the bauxite residue with CO_2_ at 30 bar of CO_2_‐partial pressure (*P*_CO2_) and a temperature of 150 °C. The formation of large aggregates of particles was observed after neutralisation, and this aggregation affected the efficiency of the subsequent leaching process with diluted H_2_SO_4_. Part of the acid was consumed by the chemical conversion of calcite, CaCO_3_, to bassanite, Ca_2_(SO_4_)_2_⋅H_2_O, and silicate compounds. The extraction of REEs was, on average, less than 30 %, so that most of the metals remained unreacted in the residue after acid leaching due to the formation of a passivating carbonate layer that surrounded the host mineral particles.^[41]^ Although the leaching process could have been benefited from replacing sulphuric acid by another acid such as hydrochloric acid or methanesulfonic acid (MSA) for leaching the neutralised material,^[42]^ acid leaching could have also released the CO_2_ immobilised in the carbonate phases formed in the bauxite residue. However, the CO_2_ released by the decomposition of carbonate minerals in acidic conditions could then be recirculated for use in the neutralisation stage – principle of Regenerate Reagents.

Petrakova *et al*. reported the utilisation of H_2_CO_3_ combined with NaHCO_3_ for the extraction of scandium from bauxite residue.^[43]^ The proposed methodology took advantage of the high alkalinity of bauxite residue to extract scandium selectively from the solid matrix in alkaline conditions. After a desilication pretreatment step, NaHCO_3_ was selected as leaching medium due to a higher solubility for Sc(OH)_3_ in a NaHCO_3_ solution compared to a Na_2_CO_3_ solution.^[44]^ CO_2_ was introduced into an autoclave reactor at relatively low partial pressure (up to 6.1 bar) and 60 °C, which allows to shift the Sc(OH)_3_ dissolution reaction to the dissolved products (Equation (5)), and improve the transfer of scandium to the leach liquor (Equation (6)). About 125 g dm^−3^ of NaHCO_3_ was required for a maximum scandium extraction of ≈27 %.

(5) $$\mathrm{Sc}{\left(\mathrm{OH}\right)}_{3}+\mathrm{NaHC}O_{3}\to \mathrm{Na}\left(\mathrm{Sc}{\left(CO_{3}\right)}_{2}\right)+\mathrm{NaOH}+2H_{2}O$$

(6) $$\mathrm{NaOH}+{\mathrm{CO}}_{2}\to \mathrm{NaHC}O_{3}$$

#### 2.1.3 Purification of Lithium Carbonate

Aqueous CO_2_ has been used for purification of Li_2_CO_3_ by a process that considers the formation of soluble LiHCO_3_ from lithium‐containing brines in the presence of CO_2_ (principle Use Benign Chemicals), and the subsequent recrystallisation of Li_2_CO_3_.^[45]^ After dissolution of lithium as LiHCO_3_, CO_2_ is then partially or completely removed by raising the solution temperature, and the removal of CO_2_ induces the precipitation of pure Li_2_CO_3_. Part of the solution can be returned to the bicarbonation reaction zone to enhance the economics of the process, while the final solution can be neutralised to yield technical‐grade lithium carbonate. In the process, insoluble impurities such as iron, magnesium and calcium can be removed in the bicarbonation process either by filtration or centrifugation, whereas soluble divalent or trivalent ions, such as magnesium, calcium and iron ions, can be absorbed by selective ion exchange. Similarly, Yi *et al*. investigated the direct carbonation process of Li_2_CO_3_ slurries with aqueous CO_2_ for the formation of more soluble LiHCO_3_ at pressures between 0.1 and 0.6 bar.^[46]^ Their results indicated that the dissolution rate of Li_2_CO_3_ increased with the increase in CO_2_ pressure inside the reactor, CO_2_ flow rate, agitation speed, or decrease of temperature. Yet, multiple carbonations cycles were needed to improve the solubilisation of lithium and the removal of impurities. The recovery of CO_2_ and production of pure Li_2_CO_3_ precipitate could be accomplished by heating the purified LiHCO_3_ solution under vacuum conditions.

Han *et al*. studied the heterogenous precipitation of Li_2_CO_3_ from Li_2_SO_4_ solution by bubbling CO_2_ directly into the aqueous phase.^[47]^ The initial pH of the solution (before carbonation) was in the range 9.2–11.9. When introducing CO_2_ gas into the solution, equilibrium was attained at a pH value of about 8.0, indicating the end of the carbonation period. Small particle sizes were obtained at high rotation speeds due to the supersaturation created by the formation of small gas bubbles. On the other hand, larger particles precipitated when the temperature was increased.

**Figure 3:**
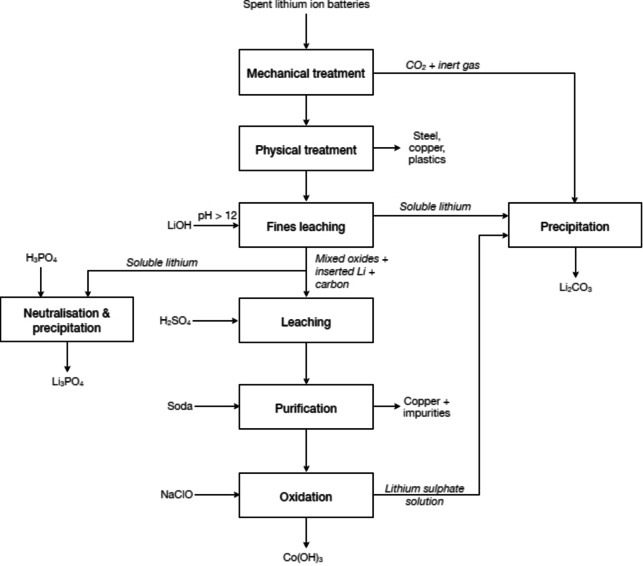
Recupyl process for recycling spent lithium‐ion batteries (adapted from [48]).

The Recupyl process (France) is a method for treating all types of lithium anode cells and batteries by hydrometallurgy at room temperature.^[48]^ As described in Figure 3, the mechanical processing of spent batteries is conducted within an inert atmosphere to mitigate the reactivity of lithium. Subsequently, plastics, steel, and copper components undergo physical separation methods. The resulting fine powder, obtained through screening, is then suspended in agitated water to facilitate subsequent leaching and hydrolysis steps. Following filtration of the hydrolysed solution, an alkaline lithium salt solution is generated alongside a suspension containing metal oxides and carbonaceous material. Lithium precipitation occurs in the form of Li_2_CO_3_ utilising CO_2_ derived from the mechanical processing. Concurrently, the suspension of metal oxides undergoes dissolution in sulfuric acid. Copper is subsequently cemented out through interaction with steel shots. The purified solution undergoes oxidation with sodium hypochlorite (NaClO) to induce the precipitation of cobalt(III) hydroxide, Co(OH)_3_, after which cobalt is separated via electrolysis. The residual lithium within the solution is precipitated through the introduction of CO_2_ gas. As the process focuses on the recycling of spent batteries, makes use of a benign solvent, and enable the recovery of different products, it complies with the principles Use Benign Chemicals, Safely Dispose of Potentially Harmful Elements, Prevent Waste, and Maximise Mass Efficiency.

### 2.2 Ammonium (Hydrogen)Carbonate Salts

Ammonium carbonate (NH_4_)_2_CO_3_ and/or ammonium hydrogencarbonate (NH_4_)HCO_3_ can be easily formed by mixing CO_2_ with an aqueous ammonia solution (NH_3,aq_) (Equations (7) and (8), respectively), which are among the cheapest chemicals and they can be recycled in the same process with minimal environmental impact; thus complying with the principles Regenerate Reagents, Prevent Waste, and Use Benign Chemicals.^[49]^ The combination of ammonia with ammonium carbonate is frequently reported in the literature as a leaching medium for the dissolution of target metals (commonly known as the AAC process). These type of carbonate salts have also been applied as precipitating reagents.

(7) $$2\;\;NH_{3}+H_{2}O+CO_{2}\to {{\left(NH_{4}\right)}_{2}\mathrm{CO}}_{3}$$

(8) $$NH_{3}+H_{2}O+CO_{2}\to {\left(NH_{4}\right)\mathrm{HCO}}_{3}$$

#### 2.2.1 Ammonium (Hydrogen)Carbonate as Leaching Solutions

##### 2.2.1.1 Zinc

**Figure 4:**
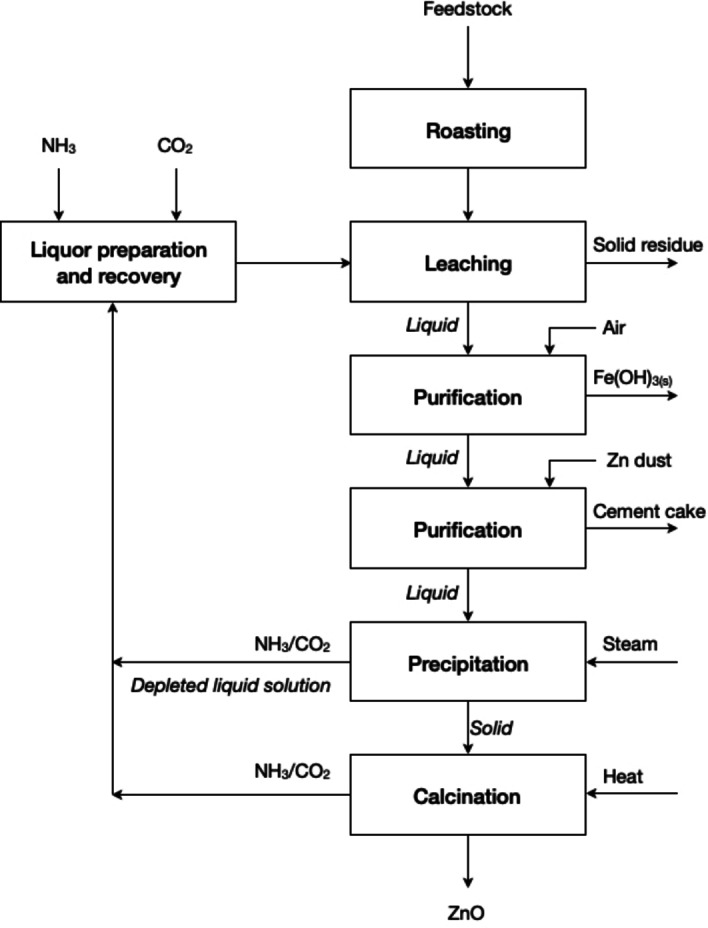
Process flowsheet of the Schnabel process (adapted from [49] with permission from Taylor & Francis, copyright 2006).

The so‐called “Schnabel process”, which originates from the 1880s, considers a combination of NH_3_ and (NH_4_)_2_CO_3_ as medium for the extraction of zinc.^[49]^ Although the process has underwent several modifications over time, the general flowsheet still exhibits the same basic unit processes (see Figure 4) that can be summarised as follows: (1) roasting; (2) leaching with mixed solution of NH_3_ and (NH_4_)_2_CO_3_; (3) purification of the pregnant leach solution by cementation with zinc powder; (4) air oxidation to precipitate iron as Fe(OH)_3_; (5) process to recover NH_3_; and (6) precipitation of zinc as basic zinc carbonate by steam heating. The chemical composition of pure basic zinc carbonate is [ZnCO_3_]_2_⋅[Zn(OH)_2_], but the chemical formula of this compound is often written in a simplified form as ZnCO_3_ because the precipitate comprises mainly ZnCO_3_ with variable amounts of Zn(OH)_2_. During steam heating, (NH_4_)_2_CO_3_ is decomposed into NH_3_ and CO_2_; (7) recovery of NH_3_ and CO_2_ – the principle of Regenerate Reagents in circular hydrometallurgy – and recycling of the lixiviant to the depleted solution; and (8) calcination of the basic zinc carbonate to ZnO.

**Figure 5:**
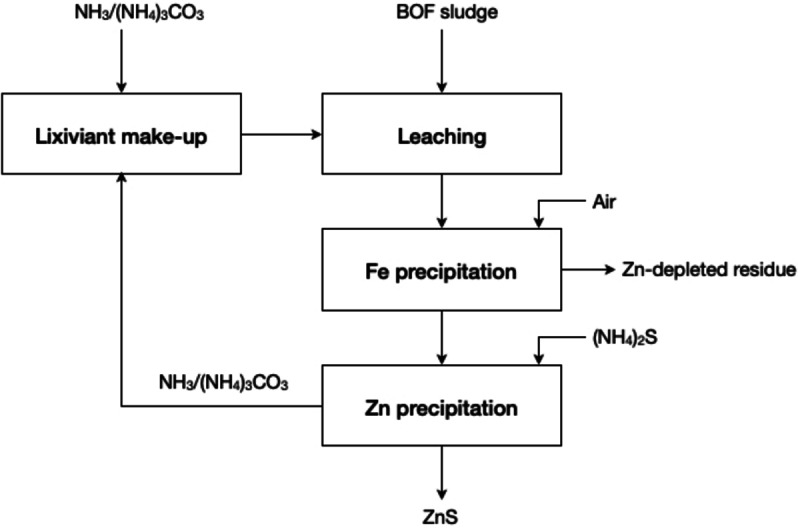
Proposed process flowsheet for extracting zinc from BOF sludge (adapted from [50] with permission from Springer Nature, copyright 2020)

Compared to acid leaching, ammonia ammonium carbonate leaching offers a higher selectivity for solubilisation of zinc over most common impurities in zinc ores, such as lead, iron, manganese, and silicon, due to the formation of the soluble zinc ammine complex [Zn(NH_3_)_4_]^2+^.[^50^, ^51^] However, an excess of NH_3_ is required to keep the zinc in solution as the ammine complex. A flowsheet for the selective removal of zinc from basic oxygen furnace (BOF) sludge using AAC leaching (Figure 5), followed by iron and zinc precipitation with the introduction of air and ammonium sulfide (NH_4_)S, respectively, was suggested by Rodriguez‐Rodriguez *et al*.^[50]^ After precipitation, and following the principles of reagent regeneration and waste prevention, the AAC solution could be recycled for reuse as lixiviant.

**Figure 6:**
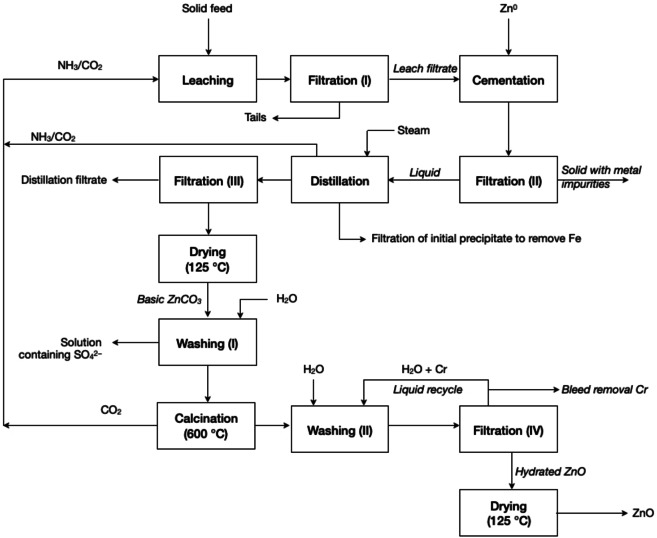
Proposed process flowsheet for producing ZnO from steelmaking flue dust (adapted from [52])

A patented process technology for recovering zinc from steelmaking dust suggest that leaching must be carried out at temperatures lower than 60 °C or under pressurised conditions to avoid the undesirable evolution of NH_3_ and CO_2_, which can lead to the premature precipitation of zinc.[^52^, ^53^] A process patented by Peters, described in Figure 6, comprises leaching zinc‐rich flue dust with aqueous NH_3_ and CO_2_ to dissolve zinc and unwanted impurities. High concentrations of free NH_3_ increased the total leaching capacity with respect to zinc, by a tendency to form metal ammine complexes. On the other hand, an increase in CO_2_ can lead to the formation of insoluble carbonates. The amount of NH_3_ added should be high enough for the sum of NH_3_ and ammonium to be 3–7 mol L^−1^, whereas the addition of CO_2_ should be sufficient for the sum of the carbonate and hydrogen carbonate ions to be 1–3 mol L^−1^ (a ratio NH_3_ to CO_2_ of 3 : 1 was suggested). The filtered pregnant leach solution is then subjected to cementation with pure zinc to remove copper, cadmium and lead impurities. Depending on the lead concentration, the filter cake (tails) from the first filtration can be rich in lead(II) carbonate, and be particular suitable for recycling by secondary lead smelters. Alternatively, the lead(II) carbonate may be recycled to produce PbO and CO_2_, which then can be used to regenerate the AAC solution, i. e. principle Regenerate Reagents. On the other hand, the solid fraction obtained from the second filtration can be rich in cadmium due to the reaction with zinc powder or dust, which can serve as input material for cadmium refineries. Steam distillation is carried out afterward on the liquid stream obtained from the second filtration for precipitating and removing iron. NH_3_ and CO_2_ are recovered from the distillation to be reused during leaching. The liquid stream is further filtrated leaving behind basic zinc carbonate. This product is then washed to remove soluble sulphates, sodium and potassium contaminants, and calcined at 250–600 °C to form ZnO. The calcined product is washed with water to remove chromium and further dried to produce high purity ZnO. Aeration after leaching can oxidise the remaining ferrous iron present and precipitate it as iron(III) hydroxides.^[54]^ Solid waste streams can be further reacted with an alkaline solution for its neutralisation and safe disposal, whereas the leaching liquor can be mixed with the liquid waste of the ammoniacal process to increase its pH and to enhance the recyclability of NH_3_.^[55]^ Overall, the process complies with the principles Use Benign Chemicals, Safely Dispose of Potentially Harmful Elements, and Regenerate Reagents.

Varga and Torok reported the utilisation of the AAC solution for the extraction of zinc from electrical arc furnace (EAF) dust, and the precipitation of ZnCO_3_ by introducing CO_2_, followed by steam distillation.^[56]^ Ruiz *et al*. reported that the (NH_4_)_2_CO_3_ concentration (60–200 g L^−1^) had hardly any influence on the leaching of zinc, and recovery yields of about 45 % were obtained. It was reported that the solution required a temperature of 80–90 °C to expel the NH_3_ and CO_2_, with the gases used to reform the lixiviant.^[57]^ Wylock *et al*. developed a model to predict the time evolution of the pH during the injection of CO_2_ gas in aqueous solutions of NH_3_ and (NH_4_)_2_CO_3_ during the leaching of Waelz oxides.^[58]^

**Figure 7:**
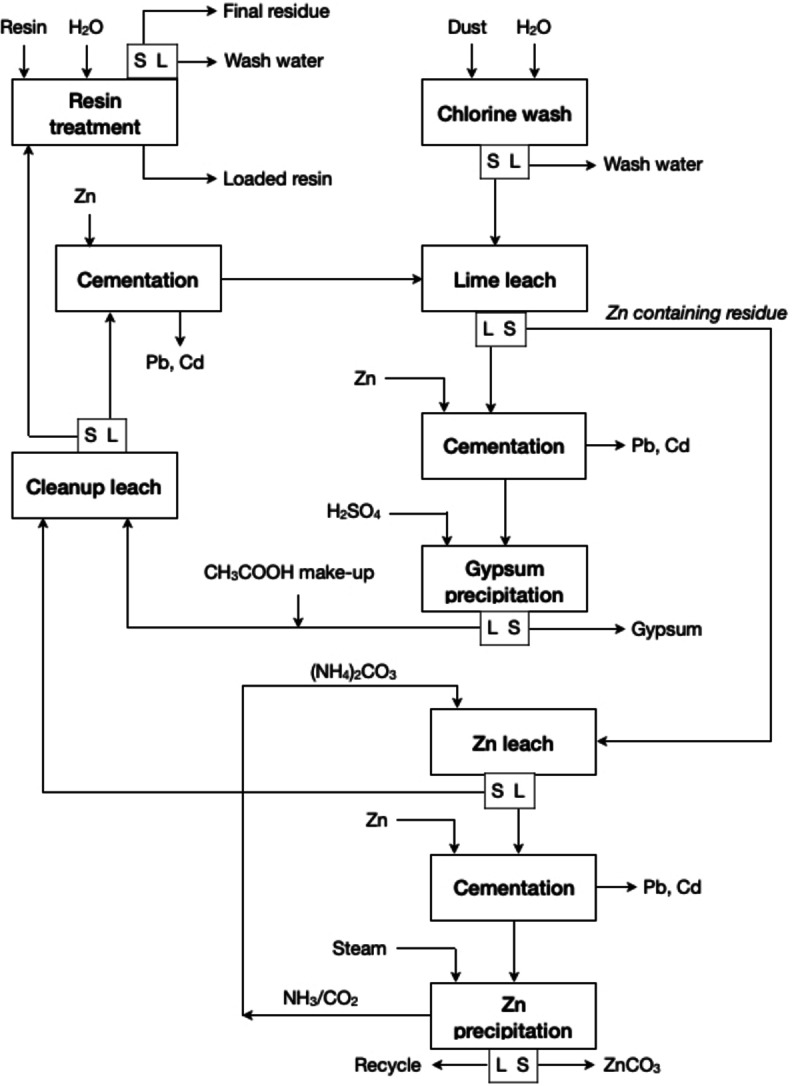
UBC‐Chaparral Process for producing ZnCO_3_ from EAF dust (adapted from [59] with permission from Elsevier, copyright 1990).

The UBC‐Chaparral process combines acetic acid (CH_3_COOH) and AAC leaching for the extraction of calcium and zinc, respectively, from EAF dust, as described in Figure 7.^[59]^ To avoid the chemical transformation of CaO into CaCO_3_, the free CaO is removed before the zinc leach with a mixture of NH_3_ and (NH_4_)_2_CO_3_. The CaO and CaCO_3_ in the EAF dust react with CH_3_COOH to form calcium acetate, Ca(OAc)_2_.The zinc acetate, Zn(OAc)_2_, formed by the reaction between ZnO and CH_3_COOH will further react with the CaO according to Equation (9). Leaching involves adding EAF dust at a concentration of 3 mol L^−1^ CH_3_COOH solution close to the boiling point of the solution. Lead and calcium can be removed from the leachate by cementation with zinc powder. Calcium can be removed by addition of H_2_SO_4_, precipitating gypsum and regenerating CH_3_COOH. The zinc‐rich residue is then leached with a mixture of NH_3_ and (NH_4_)_2_CO_3_, after which the zinc is precipitated as basic zinc carbonate by steam stripping. The final residue is treated with a very acidic cation exchanger in H^+^ form by the resin‐in‐pulp (RIP) method to dissolve the lead. Eventually, the release of NH_3_ and CO_2_ from zinc precipitation can be recycled to be used as make‐up for the regeneration of the (NH_4_)_2_CO_3_.

(9) $$\mathrm{CaO}+\mathrm{Zn}{\left(\mathrm{OAc}\right)}_{2}+H_{2}O\to \mathrm{Ca}{\left(\mathrm{OAc}\right)}_{2}+\mathrm{Zn}{\left(\mathrm{OH}\right)}_{2}$$

##### 2.2.1.2 Manganese

The United States Bureau of Mines developed a process based on carbamate leaching to recover manganese from BOF slags, which corresponds essentially to the AAC leaching process since ammonium carbamate (NH_3_CO_2_NH_4_) reacts in an irreversibly manner with water with formation of (NH_4_)_2_CO_3_ (Equation (10)).^[60]^ After a pretreatment high‐temperature reduction process with hydrogen gas, and under optimum conditions, carbamate leaching allowed to dissolve about 80 % of the manganese and 50 % of iron initially present in the slag. These metals were recovered from the pregnant leach solution as a mixture of carbonate salts by heating and expelling ammonia gas.

(10) $$NH_{3}CO_{2}NH_{4}+H_{2}O\to {{\left(NH_{4}\right)}_{2}\mathrm{CO}}_{3}$$

Katsiapi *et al*. described the recovery of cobalt from a mixed cobalt‐manganese hydroxide precipitate, which was obtained from sulphate leach liquors of nickel oxide ore.^[61]^ Different NH_3_/(NH_4_)_2_CO_3_ and solid‐to‐liquid (S/L) ratios were investigated at atmospheric conditions during a period of 5 h. Cobalt leaching yields varied between 85 % and 93 %, while ≈0.05 % of manganese was also co‐dissolved. Based on Yate′s and variance analysis, a 200 g/200 g ratio for NH_3_/(NH_4_)_2_CO_3_ and 10 % solid was found to be the optimum condition to achieve maximum cobalt extraction. Similarly to zinc, cobalt also forms a stable ammine complex in solution [Co(NH_3_)_6_]^2+^, whereas manganese tends to form hydroxides or oxides (Mn_3_O_4_, Mn_2_O_3_). However, a small portion of manganese can still remain soluble in the solution, re‐precipitating as MnCO_3_.

##### 2.2.1.3 Rare‐Earth Elements, Thorium and Uranium

The solubility of the REEs in solutions of (NH_4_)_2_CO_3_ was studied by De Vasconcellos *et al*., who considered a sample described as “low cerium carbonate”.^[62]^ A concentration of 200 g L^−1^ (NH_4_)_2_CO_3_ was used as lixiviant. After 10–30 min of mixing, it was observed that the heavy REEs (from Gd until Lu, plus Y) tend to be more soluble than the light REEs (from La until Eu, plus Sc). Also, when the (NH_4_)_2_CO_3_ concentration was increased from 50 to 400 g L^−1^, it was observed that the solubilisation of REEs increased as a function of their atomic number. After leaching with the (NH_4_)_2_CO_3_ solution, the filtered leach solution was treated directly with oxalic acid to precipitate yttrium(III) oxalate, which was calcined to yttrium(III) oxide. Alternatively, the solution was treated directly with hydrogen peroxide (H_2_O_2_) to produce a peroxycarbonate of REEs that can be calcined to the oxide and, in this case, cerium, praseodymium and neodymium peroxides can be directly precipitated and separated from the yttrium solution.

Abdel‐Rehim studied the carbonate leaching of the hydroxide solid residue obtained after alkaline leaching of monazite mineral.^[63]^ An Egyptian monazite sample was preprocessed by alkaline leaching in a ball‐mill autoclave. This was followed by selective separation of thorium and uranium from the REEs by autoclave leaching of the hydroxide solid residue obtained with an AAC solution. The method is based on the tendency of thorium and uranium to form soluble carbonate complexes with (NH_4_)_2_CO_3_, whereas the REEs form less‐soluble double carbonates. More than 95 % of the thorium and uranium were recovered in an autoclave of 1–2 h of leaching under the optimum conditions (70–80 °C, 30 % (NH_4_)_2_CO_3_ solution). Most of the REEs remain unreactive in the solid residue after the ammoniacal leaching.

#### 2.2.2 Ammonium (Hydrogen)Carbonate as Precipitating Reagents

##### 2.2.2.1 Manganese

**Figure 8:**
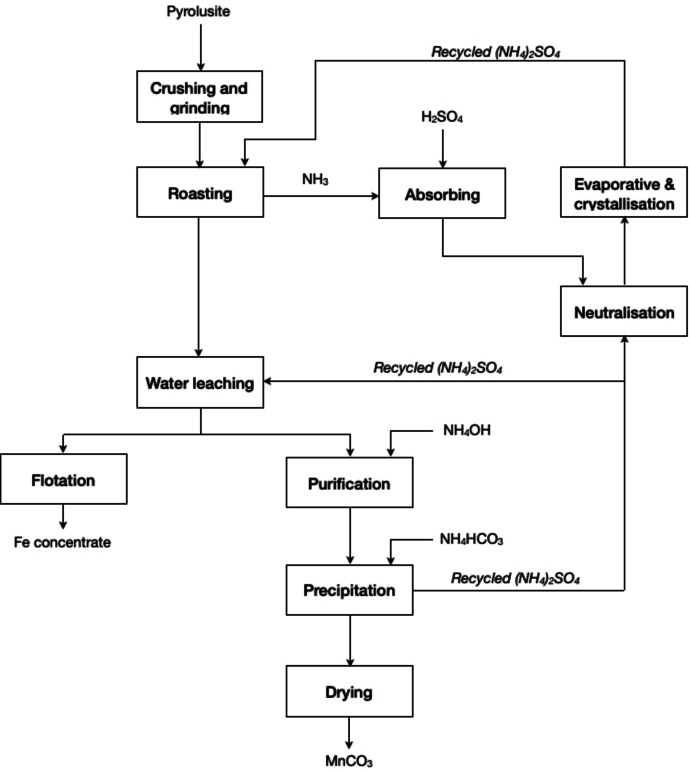
Process flowsheet for selective extraction and recovery of manganese from pyrolusite by (NH_4_)_2_SO_4_ roasting‐water leaching process (adapted from [64] with permission from Elsevier, copyright 2023).

Ju *et al*. developed an efficient and clean method for selective extraction and recovery of manganese from pyrolusite mineral (see Figure 8).^[64]^ The process entails the conversion of manganese into MnSO_4_ by sulphation roasting with (NH_4_)_2_SO_4_, followed by water leaching to solubilise the MnSO_4_. After a purification treatment, and under optimum conditions, high‐grade MnCO_3_ was produced via precipitation using NH_4_HCO_3_ at 70 °C for 60 min. The precipitation of manganese took place at a pH value of 6.0 when its concentration was about 0.5 mol L^−1^ in solution, whereas at lower concentration a solution pH of 8.5 was required to achieve complete conversion. The process allowed to recover about 95 % of the manganese, while about 94 % of the NH_3_ released during roasting could be converted to (NH_4_)_2_SO_4_ solution by absorption in dilute H_2_SO_4_ solution. Additionally, because the purification solution makes use of ammonia for pH adjustment, the filtrate from the precipitation of manganese is also an (NH_4_)_2_SO_4_ solution. These two parts of the (NH_4_)_2_SO_4_ solution can be regenerated into (NH_4_)_2_SO_4_ products through evaporation and crystallisation. This process complies with the principles Reduce Chemical Diversity, Use Benign Chemicals, and Regenerate Reagents.

**Figure 9:**
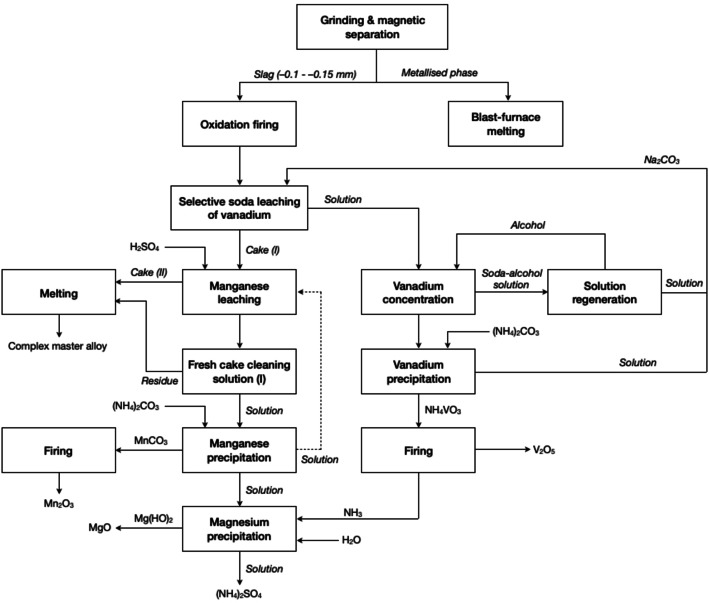
Process flowsheet for comprehensive treatment of manganese vanadium slags (adapted from [65] with permission from Springer Nature, copyright 2019).

Krasheninin *et al*. reported a process flowsheet for the comprehensive treatment of Mn/V‐rich converter slags.^[65]^ The suggested process consists of two stages (see Figure 9): (1) extraction of vanadium with a solution of Na_2_CO_3_, and (2) extraction of manganese with dilute H_2_SO_4_. In the first stage, the oxidation firing slag is refined and cleaned from metal inclusions without any additives. Vanadium is selectively leached from the slag using a solution of calcined soda, and precipitated in a subsequent step by addition of (NH_4_)_2_CO_3_. Vanadium is further refined at high temperature for producing high‐purity vanadium pentoxide (99.8 % V_2_O_5_). Ammonia is recovered from the refining process, which is used for the precipitation of magnesium. In the second stage, after extracting vanadium, magnesium remains in the solid residue. A solution of H_2_SO_4_ is then used for extracting manganese from the residue, which is later on precipitated by addition of (NH_4_)_2_CO_3_.

##### 2.2.2.2 Rare‐Earth Elements

A process has been reported for precipitating and separating REE compounds from a REE‐containing liquid using NH_4_HCO_3_ as precipitating reagent.^[66]^ The precipitation yield can reach more than 95 %, and the purity of the REE products can be over 92 %. Spiridigliozzi *et al*. studied the precipitation of selected REEs (Sm, Gd, Pr, Ce) with (NH_4_)_2_CO_3_, at room temperature for the synthesis of nanometric powders to be used as materials for intermediate temperature solid oxide fuel cells.^[67]^ Rychkov *et al*. reported the dissolution of REEs from phosphogypsum by H_2_SO_4_ leaching and further precipitation with NH_4_HCO_3_.^[68]^ De Vasconcellos *et al*. studied the solubilisation of REEs by solutions of (NH_4_)_2_CO_3_ and (NH_4_)_2_CO_3_/NH_4_OH. The latter showed better solubility than when solely (NH_4_)_2_CO_3_ was used as lixiviant.^[69]^

##### 2.2.2.3 Lithium

A process patented by Xinjiang Non‐Ferrous Metal Institute (P.R. China) uses (NH_4_)_2_CO_3_ as a precipitating reagent to recover Li_2_CO_3_ from sulphate solutions. Compared with the utilisation of Na_2_CO_3_, the proposed method does not require complex equipment but it does require a sedimentation operation, including filtration, washing and drying, for purification at temperatures between 70–100 °C. Similar recovery yields and product quality can be obtained in the presence of the ammonium salt. According to the authors, the method also allows to recover (NH_4_)_2_SO_4_ as a by‐product that can be sold as fertiliser.^[70]^

Nguyen and Lee investigated the recovery of Li_2_CO_3_ from the dust produced during the smelting reduction of spent lithium‐ion batteries.^[71]^ Na_2_CO_3_ was a more effective precipitating reagent than (NH_4_)_2_CO_3_ due to the hydrolysis reaction of dissolved ammonium into ammonia and hydrogen ions. The addition of acetone and ethanol enabled a significant improvement in the carbonation of lithium, due to the reduction of the dielectric constant of the solution.

##### 2.2.2.4 Uranium

The precipitation of uranium in the form of ammonium uranyl carbonate (AUC) in the presence of (NH_4_)_2_CO_3_ solution has also been investigated. AUC is produced by the combination of uranyl nitrate and (NH_4_)_2_CO_3_ at specific pH and temperature. It was also prepared by passing NH_3_ and CO_2_ gases into uranyl nitrate solution.[^72^, ^73^] The predominant mechanism is given by a reaction with second‐order kinetics (Equation (11)).^[74]^ Kim *et al*. studied the precipitation of AUC to determine the feasibility of recycling (NH_4_)_2_CO_3_ solution by modifying the flow rate in order to avoid formation of agglomerated crystals.^[75]^

(11) $$UO_{2}{\left(NO_{3}\right)}_{2}\cdot 6H_{2}O_{\left(aq\right)}+6NH_{3\left(g\right)}+3CO_{2\left(g\right)}\to {\left(NH_{4}\right)}_{4}UO_{2}{\left(CO_{3}\right)}_{3\left(s\right)}+2\;\;NH_{4}NO_{3\left(aq\right)}+3H_{2}O_{\left(l\right)}$$

##### 2.2.2.5 Aluminium

Ammonium aluminium carbonate hydroxide [NH_4_Al(OH)_2_CO_3_, or AACH], also known as ammonium dawsonite, has been recognised as one of the promising precursors for preparing *α*‐Al_2_O_3_ powder. Various methods can be used to synthesise AACH, but the main one remains the precipitation of aluminium salts [Al(NO_3_)_3_, (NH_4_)Al(SO_4_)_2_, or AlCl_3_] in aqueous phase with (NH_4_)_2_CO_3_ or NH_4_HCO_3_.[^76^, ^77^, ^78^, ^79^] Influence of several variables on the product properties have been studied, such as reactants, contacting mode, pH, and molar ratios.[^76^, ^80^, ^81^, ^82^, ^83^, ^84^, ^85^, ^86^, ^87^]

##### 2.2.2.6 Magnesium

**Figure 10:**
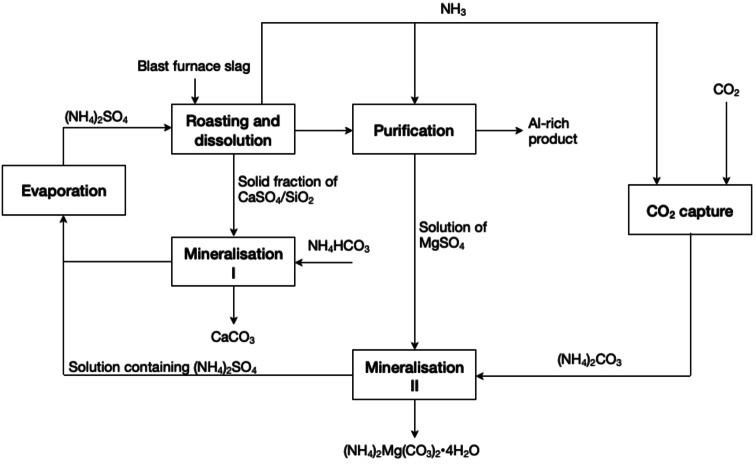
Process flowsheet of the mineral carbonation process with blast furnace (BF) slag (adapted from [31] with permission from Elsevier, copyright 2017).

Hu *et al*. reported the carbonation of calcium and magnesium from blast furnace (BF) slags by adding (NH_4_)_2_CO_3_ and NH_4_HCO_3_ solutions, respectively.^[31]^ In a first stage, (NH_4_)_2_SO_4_ was used for extracting calcium, magnesium and aluminium from the BF slag in a process that combines low‐temperature roasting (<400 °C) with water leaching (Figure 10). The process considers the reutilisation of the NH_3_ gas resulting from the roasting process (Equation (12)) for adjusting the pH (up to 5.5) of the acidic leaching solution of the roasted slag. The increase in pH allowed to precipitate most of the aluminium, leaving behind a leaching solution enriched in magnesium and a solid residue containing most of the calcium. Then, both the liquid and the solid are subjected to carbonation in the presence of (NH_4_)_2_CO_3_ and NH_4_HCO_3_ solutions (Equation (13)), respectively. The method allowed to convert more than 90 % of calcium (into CaCO_3_) and magnesium (into (NH_4_)_2_Mg(CO_3_)_2_⋅4H_2_O) present in the BF slag. NH_3_ could also be further recovered from the magnesium carbonate product by heating it at 100–200 °C, *i.e*. the principle Regenerate Reagents.

(12) $${\left(\mathrm{Ca},\mathrm{Mg}\right)}_{x}{\mathrm{Si}}_{y}Ox+2y+zH_{2z\left(s\right)}+x\left(NH_{4}\right)SO_{4(s)}\to x\left(\mathrm{Ca},\mathrm{Mg}\right)SO_{4(s)}+y\mathrm{Si}O_{2(s)}+\left(z+x\right)H_{2}O_{\left(g\right)}+2xNH_{3\left(g\right)}$$

(13) $$\left(Ca,Mg\right)SO_{4\left(s\right)}+CO_{2\left(g\right)}+H_{2}O_{\left(l\right)}+2NH_{3\left(g\right)}\to \left(Ca,Mg\right)CO_{3\left(s\right)}+{\left(NH_{4}\right)}_{2}SO_{4\left(aq\right)}$$

**Figure 11:**
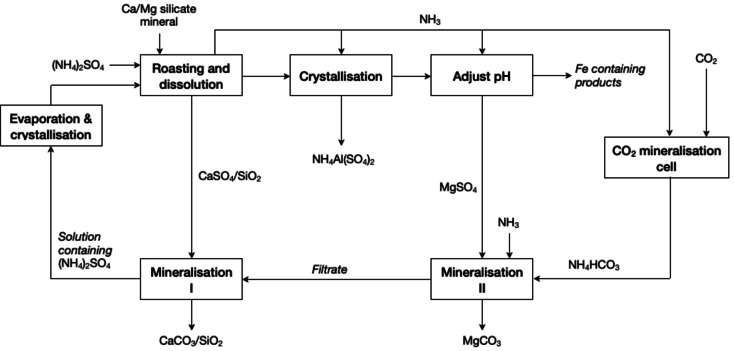
Process flowsheet of the mineral carbonation process of calcium/magnesium silicate minerals (adapted from [88] with permission from Elsevier, copyright 2018).

The process was further optimised by Liu *et al*. who reported the utilisation of ammonium hydrogensulphate ((NH_4_)HSO_4_) as lixiviant and make use of the gaseous NH_3_ for CO_2_ capturing in a mineralisation cell, which simultaneously generate NH_4_HCO_3_ and electricity.^[88]^ As it is described in Figure 11, carbonation was carried out in both the solid residue and the leaching solution by using a mixed solution containing NH_4_HCO_3_ and NH_3_. Aluminium was recovered from the leaching solution as NH_4_Al(SO_4_)_2_⋅12H_2_O at pH ≈ 2.5 at 20 °C. The solution′s pH was increased up to 5–6 by introducing additional NH_3_ to recover the remaining aluminium and, after adding CO_2_, magnesium was recovered as (NH_4_)_2_Mg(CO_3_)_2_⋅4H_2_O. Calcium was recovered as CaCO_3_ from the solid residue by contacting it with a magnesium carbonation mother liquor at 55 °C. Due to the recirculation of different reagents (NH_3_, (NH_4_)_2_CO_3_, (NH_4_)_2_SO_4_), the minimisation of waste generation, and the generation of electricity in a mineralisation cell, this process exemplify very well the principles Reduce Chemical Diversity, Prevent Waste, and Electrify Processes Wherever Possible.

Frolova *et al*. study the phase composition of iron oxide compounds formed during precipitation by (NH_4_)_2_CO_3_ hydrolysis products.^[89]^ The formation of iron(II) salts with (NH_4_)_2_CO_3_ can be described as follows: (1) reaction of hydrolysis of (NH_4_)_2_CO_3_ (Equation 14); (2) formation of iron(II) hydroxide or the basic ferrous salt depending on the molar ratio between the precipitating reagent and the ferrous salt (Equation 15); and (3) oxidation of the iron(II) compounds to iron(III) (either by following Equation 16 or Equation 17). The overall reaction for magnetite formation is described by Equation 18.

(14) $${\left(NH_{4}\right)}_{2}CO_{3}+H_{2}O\to 2\;\;NH_{4}OH+CO_{2}$$

(15) $$2\;\;NH_{4}OH+FeSO_{4}\to Fe{\left(OH\right)}_{2}+{\left(NH_{4}\right)}_{2}SO_{4}$$

(16) $$4Fe{\left(OH\right)}_{2}+O_{2}\vec{\leftarrow }4\alpha -FeOOH+2H_{2}O$$

(17) $$3Fe{\left(OH\right)}_{2}+{1/2O}_{2}\vec{\leftarrow }{Fe}_{3}O_{4}+3H_{2}O$$

(18) $$3{\left(NH_{4}\right)}_{2}CO_{3}+3FeSO_{4}+\;1/2O_{2}\to {Fe}_{3}O_{4}+{3\left(NH_{4}\right)}_{2}SO_{4}+3\;\;CO_{2}$$

### 2.3 Sodium (Hydrogen)Carbonate Salts

Sodium carbonate, Na_2_CO_3_, (commonly known as *soda ash*) and sodium hydrogencarbonate (also called sodium bicarbonate), NaHCO_3_, are mainly produced by the Solvay process by contacting a NaCl solution with CO_2_ in the presence of NH_3_. This is a reversible reaction forming NH_4_Cl and NaHCO_3_ (Equation (19)), which is carried out in a tower of ca. 25 m high where a downward flow of ammonia dissolved in brine contacts an upward flow of CO_2_.^[90]^ Ammonia is recovered by addition of lime (CaO), but the process is energy‐intensive and the carbonation reaction requires several cycles before completion. Alternatively, Zha *et al*. patented a process for the production of Na_2_CO_3_ (among others alkali metal (hydrogen)carbonate, such as K_2_CO_3_) from brines. The method entails: (1) introduction of CO_2_ into an aqueous solution of an alkali metal salt in contact with a water‐immiscible organic amine‐containing phase, so that the formation of an alkali metal (hydrogen)carbonate and an amine inorganic acid complex can be formed in the aqueous and organic phase, respectively; (2) separation of the organic phase from the aqueous phase; (3) eventual recovery of the alkali metal (hydrogen)carbonate from the aqueous phase; (4) conversion of the so‐recovered alkali metal hydrogencarbonate into an alkali metal carbonate; and (5) regeneration of the organic phase with an aqueous base solution.^[91]^

(19) $$Na{Cl}_{\left(aq\right)}+NH_{3\left(aq\right)}+H_{2}O_{\left(l\right)}+CO_{2\left(g\right)}\vec{\leftarrow }{NH}_{4}Cl_{\left(aq\right)}+NaH{CO}_{3\left(s\right)}$$

Aqueous solutions of sodium (hydrogen) carbonate have been studied as a mild lixiviant, as it is less corrosive than acids; thus, complying with the principle Use Benign Chemicals. It also provides an improved selectivity for the extraction of metals in alkaline conditions.

#### 2.3.1 Sodium (Hydrogen)Carbonate Salts as Leaching Reagent

##### 2.3.1.1 Tungsten

The soda ash leaching of scheelite concentrates, in an autoclave at elevated temperatures (190–225 °C), is a commercial process that is in use since the 1940s.^[92]^ The dissolution of scheelite in Na_2_CO_3_ is a reversible reaction, which can be described by Equation 20.

(20) $$Ca{WO}_{4\left(s\right)}+{Na}_{2}{CO}_{3\left(aq\right)}\vec{\leftarrow }{Na}_{2}WO_{4\left(aq\right)}+Ca{CO}_{3\left(s\right)}$$

Martins studied the kinetics of soda ash leaching of low‐grade scheelite concentrates under pressure and at different temperatures (100–200 °C).^[93]^ Short reaction times lead to formation of a passivating layer of CaCO_3_, which was later on avoided by addition of H_2_Na_2_EDTA as complexing reagent.^[94]^ The dissolution of scheelite increases with increasing temperatures. However, the selectivity of the reaction decreases when decreasing the temperature and Na_2_CO_3_ concentration. In a similar study, Cheng *et al*. investigated the extraction of tungsten from scheelite at lower temperatures (25–75 °C) and at atmospheric conditions.^[95]^ Dissolution rates were predicted by the shrinking core model and compared with dissolution rates obtained at higher temperatures (150–190 °C). Over 90 % of tungsten was extracted from the tailings within 15 days by keeping the temperature at 75 °C. In a different study, more than 80 % of tungsten was extracted from scheelite ores and concentrates by using an autoclave at pressure of 9.5 bar and 180 °C during 2 h.^[96]^ For industrial scaling and design, a Na_2_CO_3_/CaWO_4_ ratio between 3.5 and 5.0 was suggested, whereas the concentration of Na_2_CO_3_ ranged between 25 and 124 g L^−1^.

##### 2.3.1.2 Uranium

**Figure 12:**
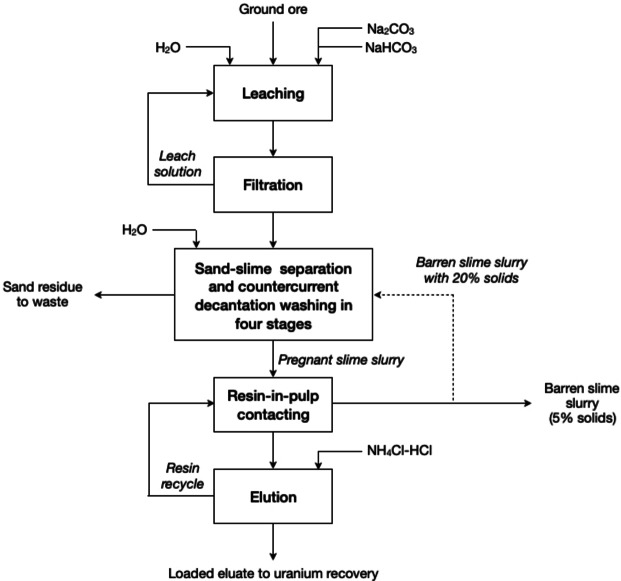
Alkaline leach processing of a subgrade calcareous uranium ore (adapted from [97]).

A process to recover uranium from calcareous uranium was developed by the United States Bureau of Mines (Figure 12).^[97]^ The process considers 50 g L^−1^ Na_2_CO_3_ and 10 g L^−1^ NaHCO_3_ as leaching solutions at 90 °C to dissolve uranium in a period of 4 h. The leach solution is recovered after solid–liquid separation of the slurry. Counter current decantation (CCD) is used to separate the sand from the slime using either water, or a combination of barren slime slurry and water as washing solution. Reuse of part of the barren slime slurry for washing would reduce the amount of fresh water needed in the upstream process, i. e. principle Close Water Loops. Uranium is recovered by the resin‐in‐pulp ion‐exchange technique, while the elution of the resin is carried out with a NH_4_Cl–HCl solution. Carbonate ions dissolve uranium by formation of soluble uranyl tricarbonato anion (Equation (21)) at a pH value around 10.^[98]^ The increase in carbonate concentration, however, leads to a reduction in the concentration of hydrogencarbonate ions, which concurrently reduces the pH buffer effect, leading to the formation of calcite (Equation (22)). An excess of hydroxide ions can lead to the precipitation of the dissolved uranium through the formation of sodium diuranate (Equation (23)), which normally occurs at pH values above 11. Therefore, an excess of HCO_3_^−^ ions is added to the system to prevent precipitation of uranium.

(21) $$CaU_{2}O_{7\left(s\right)}+4H{CO}_{3\left(aq\right)}^{-}+2{CO}_{3}^{2-}\vec{\leftarrow }2{\left[UO_{2}{\left(CO_{3}\right)}_{3}\right]}_{\left(aq\right)}^{4-}+Ca{\left(OH\right)}_{2\left(aq\right)}H_{2}O_{\left(l\right)}$$

(22) $$CaU_{2}O_{7\left(s\right)}+7{CO}_{3}^{2-}+3H_{2}O_{\left(l\right)}\vec{\leftarrow }2{\left[UO_{2}{\left(CO_{3}\right)}_{3}\right]}_{\left(aq\right)}^{4-}+CaCO_{3\left(s\right)}+6OH_{\left(aq\right)}^{-}$$

(23) $$2{\left[UO_{2}{\left(CO_{3}\right)}_{3}\right]}_{\left(aq\right)}^{4-}+14{Na}_{\left(aq\right)}^{+}+6OH_{\left(aq\right)}^{-}\vec{\leftarrow }{Na}_{2}U_{2}O_{7\left(s\right)}+6{Na}_{2}CO_{3\left(aq\right)}+3H_{2}O_{\left(l\right)}$$

##### 2.3.1.3 Vanadium

Vanadium cannot be efficiently leached from vanadium/uranium oxide‐rich minerals by a Na_2_CO_3_ solution as lixiviant, except after salt roasting that enables the conversion of less reactive vanadium compounds into water‐soluble vanadate.^[99]^ Lu *et al*. studied the extraction of vanadium from a vanadium‐rich slag using calcification roasting and Na_2_CO_3_ leaching.^[100]^ Vanadium present in the vanadium spinel coulsonite, FeV_2_O_4_, was transformed during roasting into calcium vanadate, Ca(VO_3_)_2_, which was leached with an aqueous Na_2_CO_3_ solution. Vanadium extraction from the slag yields up to 87 % under optimum conditions: 6 % CaO additive content in the roasted samples, 10 : 1 mL g^−1^ liquid–solid ratio, 80 g L^−1^ Na_2_CO_3_, and 30 min at 90 °C. A similar study on the selective separation of vanadium from calcification roasted V/Cr‐slag using a solution of NaHCO_3_ was carried out by Wen *et al*.^[101]^ Approximately, 93 % of the vanadium was effectively separated from the slag when considering a leaching solution with 80 g L^−1^ NaHCO_3_ at 100 °C for 120 min. Meanwhile, the co‐dissolution of other metals, such as chromium, iron and manganese, remained below 1 % presumably due to the continuous decomposition of NaHCO_3_, which increases the solution′s pH and reduces the extent of leaching of other metals.

The effect of a carbonating solution on the leaching of vanadium from calcium metavanadate, Ca(VO_3_)_2_, was investigated by Shi *et al*. in solutions containing Na_2_CO_3_, NaHCO_3_ and NH_4_HCO_3_.^[102]^ The carbonate leaching reaction was described in the following steps: (1) diffusion of carbonate (CO_3_^2−^) and hydrogencarbonate (HCO_3_^−^) ions from the solution to the surface of the particle; (2) CO_3_^2−^ and HCO_3_^−^ reacts with calcium metavanadate; (3) the reaction product, CaCO_3_, forms on the surface of the particle; and (4) the reaction product, the metavanadate ion VO_3_^−^, diffuses to the solution from the surface of the particle through the liquid film. CaCO_3_ particles formed on the surface of calcium metavanadate over time, but it decreased when increasing the concentration of the carbonate solution, which also allows to enhance the dissolution of vanadium. The maximum vanadium dissolution was achieved in the NaHCO_3_ solution.

Ghorbani and Montenegro, on the other hand, studied the effect of alkali carbonate–hydrogencarbonate column leaching on the dissolution of vanadium and uranium from calcium‐carbonate‐rich uranium ores.^[103]^ A new leaching index was defined to correlate the time period for process and solution consumption, which were identified as key variables for process design and scalability. Although the dissolution of thorium was almost negligible in this study, the solubility of thorium in the carbonate and hydrogencarbonate solutions was also investigated.[^104^, ^105^, ^106^]

##### 2.3.1.4 Iron and Manganese

The leaching of manganese and iron from gold‐rich tailings using Na_2_CO_3_ was studied by Mashifana and Sithole.^[107]^ About 40 % of the iron was extracted when using a leaching solution with 0.5 mol L^−1^ Na_2_CO_3_, whereas ca. 46 % of the manganese was extracted with a less concentrated solution (0.25 mol L^−1^) at room temperature and over a period of 24 h. Wu *et al*. studied pressurised alkaline self‐leaching of gold in an oxygen atmosphere as an environmentally friendly and in absence of gaseous emissions leaching process.^[108]^ The method makes use of pyrite oxidation to release the wrapped gold, which is leached by the generated thiosulphate. Pyrite is oxidised according to Equation 24 to produce metastable thiosulphate anion under oxygen atmosphere in alkaline conditions, whereas gold reacts *in situ* with the formed thiosulphate according to Equation 25.

(24) $$4\mathrm{Fe}S_{2}+7O_{2}+8OH^{-}+2H_{2}O\vec{\leftarrow }4\mathrm{Fe}{\left(\mathrm{OH}\right)}_{3}+4S_{2}O_{3}^{2-}$$

(25) $$4\mathrm{Au}+8S_{2}O_{3}^{2-}+O_{2}+2H_{2}O\vec{\leftarrow }4\mathrm{Au}{\left(S_{2}O_{3}\right)}_{2}^{3-}+4OH^{-}$$

During the oxidation process of sulphide mineral, iron oxide tends to precipitate on the surface of the parent minerals, thus reducing the oxidation reaction and gold leaching efficiency. Na_2_CO_3_ was used in this study to avoid the precipitation of iron oxide, by forming soluble iron carbonato complexes in alkaline conditions, during the concurrent dissolution of pyrite. The addition of Na_2_CO_3_ enabled the formation of the soluble iron‐carbonate complexes, [FeOHCO_3_]^−^ and [Fe(CO_3_)_2_]^2−^, preventing the formation of a passivating layer and enhancing the dissolution of pyrite.^[109]^ At high pH values (≥11) the formation of soluble iron(II)‐carbonate complexes, FeHCO_3_^+^, FeCO_3_, Fe(CO_3_)_2_^2−^, favours the oxidation of Fe(II) to Fe(III) by dissolved oxygen. This not only reduces the energy required for oxidation, but also hinders the formation of a passivating layer on the surface of pyrite. The utilisation of a benign chemical such as Na_2_CO_3_, allow to maximise the efficiency to extract gold as based on the principles of circular hydrometallurgy.

##### 2.3.1.5 Aluminium

**Figure 13:**
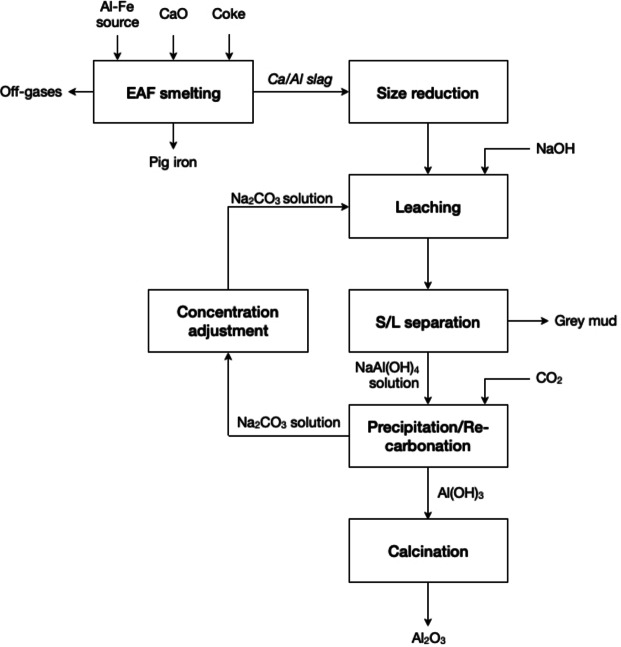
Simplified Pedersen process for the production of pig iron and aluminium oxide (adapted from [114] with permission from Elsevier, copyright 2023).

The Pedersen process was developed early in the 20th century for the hydrometallurgical extraction of aluminium from calcium aluminate slags and/or sinters in aqueous Na_2_CO_3_/NaOH solutions. However, operational and capital costs were much higher than for the Bayer process for alumina production from bauxite minerals, so that the Pedersen process is not used by industry. Nevertheless, the process was successfully operated on an industrial scale in Norway from 1925 to 1969, but it was stopped for economic reasons.^[110]^ Figure 13 describes the Pedersen process for the production of pig iron and aluminium oxide. Prior to leaching, bauxite ore (with a sufficiently high iron concentration) is reductively smelted in an electric arc furnace with fluxes, mainly lime, to recover the iron in metallic form and to produce a suitable calcium aluminate slag that can be easily solubilised in the subsequent leaching step. In the leaching step, the slag is leached with a mixed solution composed of diluted Na_2_CO_3_ and NaOH to produce a CaCO_3_ residue (called grey mud) and a pregnant sodium aluminate solution. Following a solid/liquid separation process, the pregnant leach solution enters the precipitation stage. CO_2_ is dissolved in the sodium aluminate solution. With the increase in hydrogen ion concentration, resulting from the dissolution of CO_2_, the alkaline solution is gradually neutralised, reducing the pH and triggering the decomposition of the aluminate ion, resulting in the precipitation of aluminium hydroxide.[^111^, ^112^] The use of Na_2_CO_3_ during the leaching process, however, entails the dissolution of a considerable proportion of silica together with alumina. Although, the solution can be regenerated by precipitating the alumina with CO_2_, this procedure also entails the coprecipitation of silica within the alumina product. The use of a NaOH solution, on the other hand, implies a much slower chemical reaction than with the Na_2_CO_3_ solution, but the dissolution of silica is also lower. The combination of Na_2_CO_3_ with a small volume of a NaOH solutions does not retard the dissolution of alumina, but it prevents the dissolution of silica.^[112]^ Konlechner *et al*. suggested temperatures between 70 and 90 °C for leaching, while the setup must consist of at least one leaching reactor and one settling reactor.^[113]^ Georgala *et al*. have verified the original claims stated in the patent of the Pedersen process by designing an optimised leaching process for calcium aluminate slags originated from alternative aluminium sources.^[114]^ They confirmed that the Na_2_CO_3_ leaching of calcium aluminate slags for the extraction of aluminium is a combination of caustification and aluminium dissolution processes, where the latter depends on the hydraulic character of calcium aluminates. Moreover, leaching with an excess of slag and prolonged leaching time leads to high aluminium extraction efficiencies (>85 %) independently of the leaching temperature. The co‐dissolution of silicon is temperature‐dependent, but low operational temperatures have a beneficial effect on the efficiency of the overall process.

In a study that compared the Pederson with the Bayer process, the Pedersen process showed a better utilisation of the bauxite mineral by coproduction of pig iron, but for the same reason its energy consumption is higher. Furthermore, the Pedersen process entailed twice as much of solid residue compared to the Bayer process.^[115]^ Yet, the study ignored differences between the remaining residues and its management, and it did not consider the toxicity to humans and the environmental impact. Notice that the grey mud produced in the Pedersen process has a lower alkalinity than the red mud produced by the Bayer process, so that there are less costs associated to storing and neutralisation. Moreover, the CO_2_ produced during the smelting process by burning coke can be collected and reused in the precipitation step so that the process can have a small carbon footprint.^[116]^ These environmental issues were not important at the time the original Pedersen process was developed, but they are very important nowadays. The Pedersen process complies with several of the principles of circular hydrometallurgy: (1) it makes use of CO_2_ as benign chemical; (2) waste residue are less toxic and can be dispose safely; (3) reduce chemical diversity as chemical reagents can be relatively easy to recirculate/re‐use; (4) and the final solid residue can be used for building materials; thus, having a (near) zero‐waste approach.

#### 2.3.2 Sodium (Hydrogen)Carbonate Salts as Precipitating Reagent

##### 2.3.2.1 Lead

Arai and Toguri studied the dissolution of lead sulphate, produced from scrap lead‐acid batteries by a pyrometallurgical process, in Na_2_CO_3_ solution.^[117]^ Lead is present within the batteries in the form of oxides and sulphide, as well as in elemental form. Such compounds are poorly soluble in Na_2_CO_3_ solution. All lead was converted into lead(II) sulphate (anglesite, PbSO_4_) by contacting it with H_2_SO_4_. The conversion of lead(II) sulphate into lead(II) carbonate (cerussite, PbCO_3_) by a Na_2_CO_3_ solution is thermodynamically favourable in the pH range between 6 and 10 at room temperature. Under optimum leaching conditions, lead(II) carbonate recovery could be as high as 98 % in less than about 30 min, depending on the CO_3_^2−^ ion concentration. In a similar investigation, Mbaya *et al*. considered a chemical reactor with a continuous supply of CO_2_ into a Na_2_CO_3_ solution for the selective precipitation of lead as hydrocerussite, Pb_3_(CO_3_)_2_(OH)_2_, at 25 °C and sodium lead carbonate, NaPb_2_(CO_3_)_2_OH, at temperatures higher than 45 °C.^[118]^ In a different study, it was reported that the precipitation rate increased with the first power of the Na_2_CO_3_ concentration and with the increase in temperature, but decreased when the concentration of Na_2_SO_4_ reaction product increased, i. e. at pH values above 11.^[119]^ The conversion rate was also reduced at low pH values due to the low reactivity of the HCO_3_^−^ species. The latter study also suggests that the reaction of PbSO_4_ with Na_2_CO_3_ solution is controlled by the diffusion of the Na_2_CO_3_ reactant through the growing product layer formed on the agglomerated PbSO_4_ particles.

##### 2.3.2.2 Manganese

Due to its high solubility in aqueous systems, manganese is very difficult to remove from aqueous streams and/or wastewaters; pH values above 11.0 are required for an effective removal as hydroxide. Limestone is the most common neutralising agent for acid water, but it is effective only at low manganese concentrations (<5 mg L^−1^). Silva *et al*. studied the processing of manganese‐rich mine water (with 140 mg L^−1^ Mn) with a mixed solution composed of limestone and Na_2_CO_3_.^[120]^ At Na_2_CO_3_ concentrations between 0.7–0.9 g L^−1^ in solution, it is possible to precipitate manganese to a large extent from mine water and industrial effluents at pH values above 8.0, whereas limestone induces heterogenous nucleation of MnCO_3_.

The work of Yannick *et al*. highlights the recovery of manganese from liquid effluents generated in a hydrometallurgical plant.^[121]^ Under optimum conditions (100 g L^−1^ Na_2_CO_3_, 25 °C, 1 h, pH = 8.5), ≈24 % Mn precipitated alongside 9.7 % Mg and 10.5 % Ca, all of them in the form of carbonates. Manganese dioxide (MnO_2_) was recovered through calcination, which was accompanied by a significant mass loss due to the release of CO_2_. This CO_2_ could eventually be re‐used in the precipitation stage, so that the principle Regenerate Reagents could be applied.

Jordens *et al*. studied the effect of ultrasonic power and frequency on the precipitation of MnCO_3_ in Na_2_CO_3_ solution.^[122]^ This research was mostly focused on the particle size and morphology of the precipitates. Smaller and more spherical particles were produced under the effect of ultrasonic irradiation compared to silent conditions. Likewise, the work of Kobe and Toribara compares the use of both Na_2_CO_3_ and (NH_4_)_2_CO_3_ as reagents for the precipitation of manganese from sulphate solution.^[123]^ When using a Na_2_CO_3_ solution, the precipitate became slightly brown on the surface due to the hydrolysis of Mn(OH)_2_, which absorbs free oxygen to form Mn_2_O_3_. The colour darkened when increasing the Na_2_CO_3_ concentration, but no colouration was observed when an (NH_4_)_2_CO_3_ solution was used.

##### 2.3.2.3 Aluminium

In the precipitation stage of the Pedersen process (see Section 2.3.1.5), the purified pregnant NaAl(OH)_4_ solution is treated with CO_2_ gas to form Al(OH)_3_ and, thus, the Na_2_CO_3_ solution can also be regenerated as described by Equation (26). This reaction must be carried out at temperatures below 40 °C to favour the dissolution of CO_2_ and the precipitation of aluminium as hydroxide.^[113]^

(26) $$2NaAl{\left(OH\right)}_{4\left(aq\right)}+CO_{2\left(g\right)}\to {Na}_{2}CO_{3\left(aq\right)}+2Al({OH)}_{3\left(s\right)}+H_{2}O_{\left(l\right)}$$

Marino *et al*. studied the carbonation of sodium aluminate/Na_2_CO_3_ solutions for precipitation of alumina hydrates by CO_2_ gas purging.^[124]^ The solutions were produced from the processing of calcium aluminate slags in the Pedersen process (see Figure 13). The authors highlight the importance of the carbonate ions concentration in the pregnant leach solution, as they control the precipitation reaction. According to the authors, five different precipitation stages can be distinguished during carbonation: (1) precipitation of first sodium zeolite crystals at a pH range between 12.3‐12.7, neutralisation of hydroxide ions; (2) alumina hydrate starts to precipitate while the pH drops from ca. 12.3 to about 11.0; (3) precipitation of alumina hydrates (boehmite precipitates first, followed by bayerite), aluminium recovery can reach up to 84 % after the CO_2_ bubbling has stopped (aging period). At this stage, the acidity produced by the addition of CO_2_ gas is neutralised by the alkalinity produced from the bulk precipitation of aluminium; (4) precipitation of metastable alumina hydrates induced by a sufficient aging period for enhancing aluminium recovery, pH drops from about 11.0 to 10.3; and (5) dawsonite precipitation at pH values below 10.3.

##### 2.3.2.4 Lithium

Commercial lithium is produced as lithium carbonate, lithium chloride and lithium hydroxide from various resources like brines and high‐grade lithium ores (*e.g*., spodumene, lipidolite, amblygonite and zinnwaldite). Extensive reviews on technologies for the extraction of lithium from primary and secondary sources are currently available in the literature.[^125^, ^126^] In general, after mineral beneficiation, the ore can be processed in acidic conditions (through sulfation roasting or chlorination) or alkaline conditions (through calcination). The material obtained either from the roasting, calcination or chlorination process, is crushed, milled and purified to enhance the lithium‐grade in solution. After purification, the material can be treated either with water to yield LiOH, or with HCl to produce LiCl which can be converted into Li_2_CO_3_ after adding Na_2_CO_3_. Nonetheless, due to the number of unit operations needed for recovering lithium from ores, these processes are very energy intensive and are only beneficial to process mineral resources with a high lithium grade.^[127]^ Notice that the choice between producing a specific lithium salt depends on various factors, including the specific application, market demand, and economic considerations.

**Figure 14:**
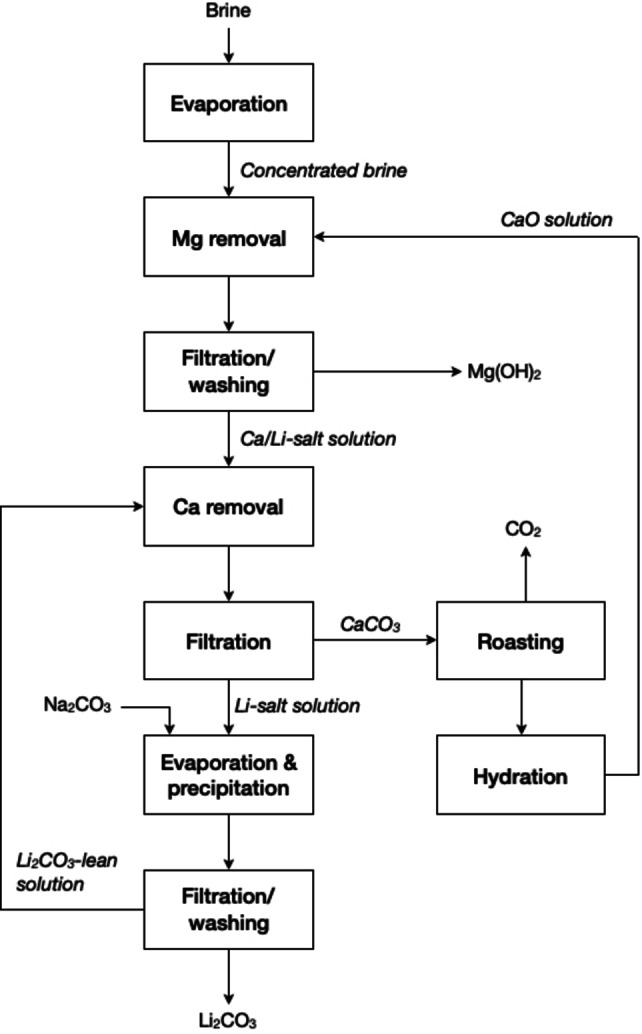
General flowsheet for the production of lithium carbonate from brine (adapted from [125] with permission from Elsevier, copyright 2014).

Lithium recovery from brines entails, initially, solar evaporation over a year in a pond to crystallise sodium, potassium and magnesium chlorides, which can be removed later on by precipitation, ion exchange and/or membrane filtration. CaCO_3_ is also removed from the concentrated brine to be roasted and then be reused to the solution of LiCl for the removal of Mg(OH)_2_, as shown in Figure 14. After calcium removal, Na_2_CO_3_ is added to the brine to precipitate lithium as Li_2_CO_3_.[^126^, ^128^, ^129^, ^130^] Eventually, the CO_2_ released from roasting could also be reused for precipitating lithium from the concentrated brine. Most often, primary lithium carbonate is redissolved and reprecipitated to reach the desired purity needed in battery grade (see Section 2.1.3).

Na_2_CO_3_ is crucial to precipitate Li_2_CO_3_ from lithium‐enriched solutions. Liu and Azimi studied the factors affecting the crystallisation of Li_2_CO_3_ from sulphate media by the addition of Na_2_CO_3_.^[131]^ Their results indicate that by increasing both the salt concentration and the temperature, better recovery efficiencies and reaction rates are obtained. The presence of sodium and calcium sulphates tend to affect both the recovery and purity of the carbonate product. The addition of seed crystals, however, facilitates crystal growth and increases the crystallisation rate. According to Han *et al*., higher crystal yields can be achieved by homogenous precipitation at 50 °C by adding Na_2_CO_3,_ which provides sufficient carbonate ions for carbonation. On the contrary, smaller crystals are obtained via direct introduction of CO_2_ into the solution (heterogenous precipitation).^[47]^ However, utilising Na_2_CO_3_ as a precipitating agent for Li_2_CO_3_ presents notable limitations. A prominent drawback involves the generation of sodium‐derived by‐products, necessitating subsequent processing or disposal unit operations. This could result in supplementary refining stages, thereby escalating process expenses. Hence, exploring alternative approaches, such as utilising CO_2_ as a carbonate source for lithium precipitation, offers potential economic and environmental benefits.

In principle, the precipitation of Li_2_CO_3_ using CO_2_ gas can be achieved in three steps: (1) formation of H_2_CO_3_ by the introduction of CO_2_ into the aqueous solution (Equations (1)–(4)), which lower the solution′s pH; (2) formation of LiHCO_3_ when the pH of the solution is above 6.3, i. e. when the HCO_3_^−^ is the dominant carbonate species ; and (3) formation of Li_2_CO_3_ when the pH exceeds 10.3, i. e. when the CO_3_^2−^ is the dominant carbonate specie. Recent studies have demonstrated that could be possible to precipitate Li_2_CO_3_ at a pH 8.0 and above using CO_2_ instead of Na_2_CO_3_ in the presence of NaOH or ammonia solution.[^132^, ^133^] Kim *et al*. describe in a short review the application of CO_2_ for Li_2_CO_3_ precipitation, and compared the utilisation of CO_2_ and Na_2_CO_3_ as carbonate sources.^[134]^ Their work also highlights the optimum operating conditions for the direct use of CO_2_ to produce micro‐sized Li_2_CO_3_ powder.

**Figure 15:**
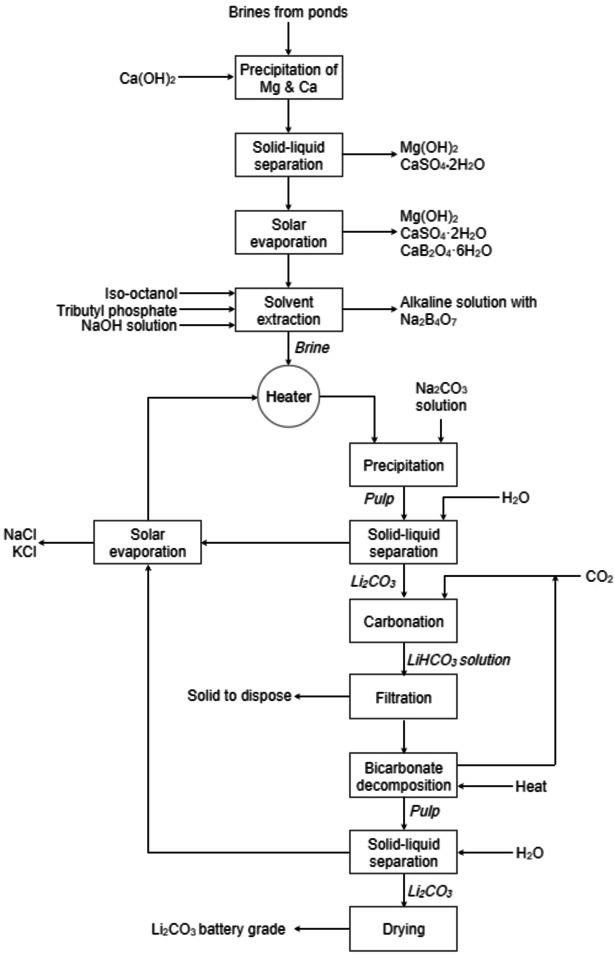
Process flowsheet for the production of lithium carbonate from brines (adapted from [135]).

A patented process for the production of Li_2_CO_3_ from natural or industrial brines considers solvent extraction as a process unit for purifying the brine and the introduction of CO_2_ for carbonation.^[135]^ The process, described in Figure 15, can be summarised as follows: (1) precipitation of magnesium by addition of Ca(OH)_2_; (2) boron removal by solvent extraction (with a mixture of TBP and *iso*‐octanol); (3) precipitation of Li_2_CO_3_ by addition of Na_2_CO_3_; (4) transformation of the Li_2_CO_3_ into LiHCO_3_ by CO_2_ (see Section 2.1.3); (5) decomposition of the LiHCO_3_ in high‐purity Li_2_CO_3_ by heating the solution. This last step enables the removal of most impurities from the Li_2_CO_3_ and the production of a battery‐grade Li_2_CO_3_.

**Figure 16:**
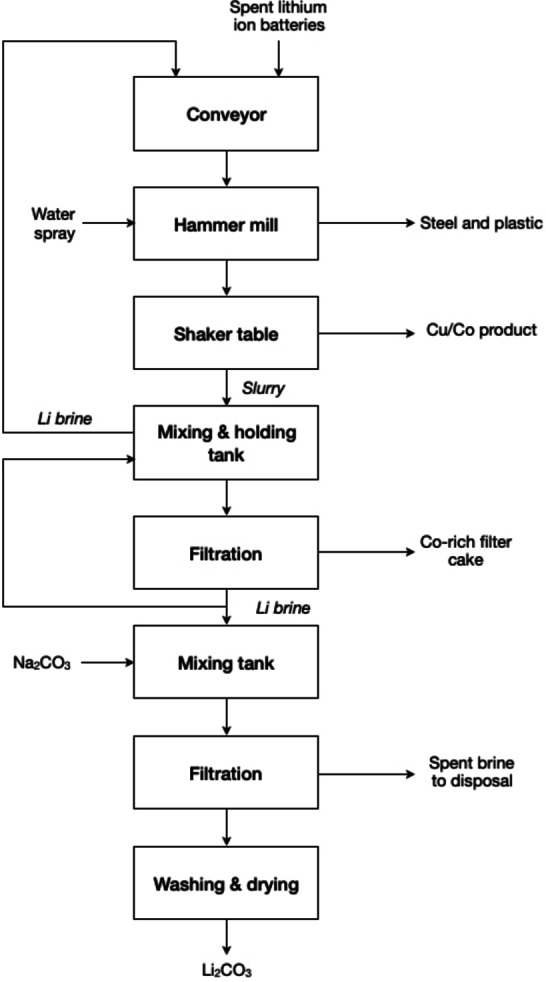
Toxco′s hydrometallurgical recycling process for spent lithium‐ion batteries (adapted from [136]).

The Toxco process (Canada) has been developed to recover lithium from a variety of spent lithium‐ion batteries (LIBs) under cryogenic conditions (in liquid nitrogen) to reduce the reactivity. The process includes mechanical and hydrometallurgical processes to recover metal values.^[136]^ The process flow diagram is depicted in Figure 16. Batteries undergo mechanical fragmentation in a caustic solution, effectively neutralising any acidic constituents and facilitating the dissolution of lithium salts. The resulting salts, upon precipitation and subsequent dewatering using a filter press, are utilised in the production of lithium carbonate. Concurrently, apart from organic compounds, hydrogen gas generated by the lithium reaction undergoes combustion at the surface of the process solution. Solid residues are directed towards cobalt recovery. Simultaneously, the large battery fragments undergo mechanical treatment while submerged in a lithium brine process solution, facilitating the retrieval of both ferrous and nonferrous metal fractions. Following the mechanical treatment, wherein lithium‐ion battery fluff, a composite of steel and plastic, is segregated from others valuable materials such as copper and cobalt. The subsequent step involves the extraction of copper. The lithium brine is subjected to treatment with Na_2_CO_3_ to yield Li_2_CO_3_. Plastic and paper constituents, which float atop the solution, are salvaged for either disposal or recycling purposes. The filtered carbonaceous residue derived from the slurry is deemed economically unviable for reutilisation or incineration. Notably, cobalt recovered from spent LIBs exhibits heightened economic value.

##### 2.3.2.5 Calcium, Magnesium, Strontium

Zhang *et al*. reported the utilisation of Na_2_CO_3_ as a pretreatment of salt‐lake brines, for the removal of calcium and magnesium, as their carbonate.^[137]^ The precipitation of both calcium and magnesium increases with increasing the carbonate concentration in solution until equilibrium was attained at a pH below 10. At a ratio Na_2_CO_3_:(Ca^2+^+Mg^2+^) of 2 : 1, the precipitation efficiencies is more than 97 %. The addition of the Na_2_CO_3_ solution works excellently for separating calcium and magnesium from the brines. Nonetheless, the required alkalinity for the subsequent process (solvent extraction coupled with CO_2_ stripping, see section 4) could only be met by addition of NaOH. This increases the consumption of chemicals.

Iwai and Toguri studied the solubilisation of strontium sulphate (celestite, SrSO_4_) in Na_2_CO_3_ solutions from thermodynamic and kinetic points of view.^[138]^ According to the authors, during the initial stage of the reaction, the conversion rate is controlled by the dissolution of SrSO_4_. The released of Sr^2+^ ions are carbonated in the presence of CO_3_^2−^ ions. The formed SrCO_3_ is deposited on the mineral surface as a dense non‐porous product layer, which hinders the diffusion of sulphate ions into the bulk solution, and limits further dissolution of the celestite mineral.

##### 2.3.2.6 Rare Earth Elements, Thorium and Uranium

The direct precipitation of thorium, REEs and uranium from a sulphate leach liquor – obtained from leaching monazite mineral with concentrated H_2_SO_4_ followed by dilution with ice water – was reported by El‐Awady *et al*.^[139]^ In the presence of 20 % Na_2_CO_3_ solution, about 64 % of the thorium and more than 80 % of selected REEs (cerium, praseodymium, neodymium, samarium) precipitated at a pH value of 0.5, whereas yttrium could be selectively recovered at pH values above 6.0. The maximum uranium precipitation (ca. 68 %) was reached at a pH value of 3.0. The filtered solution was treated directly with Na_2_CO_3_ at a pH between 6.0 and 9.0 to obtain ca. 12.5 % of Y_2_O_3_. This precipitate was purified after dissolution with diluted HCl and precipitated again at pH 3.0 in the presence of oxalic acid to reach a total recovery yield of 55 % for Y_2_O_3_.

A concentrate rich in REEs could be obtained after the consecutive treatment of phosphogypsum with solutions of NaCl and Na_2_CO_3_.[^140^, ^141^] Solutions with concentrations about 25 g L^−1^ NaCl and 60 g L^−1^ Na_2_CO_3_ were required to ensure the complete solubilisation of impurities, and effective conversion of phosphogypsum into a CaCO_3_ product. The concentrate was then contacted with diluted H_2_SO_4_, which enabled the selective solubilisation of REEs in the leach liquor to a maximum concentration of about 4.3 g L^−1^.

Gasser *et al*. studied the recovery of REEs from phosphogypsum by leaching with a 0.4 mol L^−1^ Na_2_CO_3_ solution for 120 min to obtain a mixed precipitate of calcium carbonate and REEs.^[142]^ The precipitate was then reacted with a citric acid solution allowing to achieve 87 % extraction efficiency in one leaching step under optimum conditions (L/S=5, 1 mol L^−1^ citric acid, 15 min, 85 °C). The pregnant leach solution was purified by solvent extraction using 10 % of di(2‐ethylhexyl)phosphoric acid (D2EHPA) in kerosene. The extracted REEs were stripped from the loaded organic phase by 0.5 mol L^−1^ H_2_SO_4_. The stripped solutions were further treated with 10 % oxalic acid to precipitate the REEs as their oxalates. REE oxalates can easily be converted into the corresponding oxides by a calcination step.

## 3 Carbonation Coupled With Metal Complexation

Complexation of metal ions in solution by ligands has served as a cornerstone for various processes for extraction, purification, and refinement, since metal complexes are pivotal in facilitating the separation and recovery of target metals, but also the production of high‐purity carbonates when coupled with the introduction of CO_2_. During mineral carbonation, CO_2_ is reacted with metal‐rich minerals in the presence of suitable chelating ligands to facilitate the formation of stable carbonates. By carefully selecting ligands with appropriate affinities for metal ions, it is possible to tailor the kinetics and efficiency of mineral carbonation reactions. For instance, Reynes *et al*. studied the precipitation of FeCO_3_ from an iron‐rich olivine mineral by considering a solution of NH_4_HSO_3_ as leaching medium and 2,2′‐bipyridine (bipy) as the metal complexing agent, which stabilises Fe(II) by forming the [Fe(bipy)_3_]^2+^ complex in solution.^[143]^ During leaching with 1.5 mol L^−1^ NH_4_HSO_4_ at 60 °C, about 30 % Fe(II) and 40 % Mg(II) were leached out from the matrix of the olivine mineral. The leach liquor was then subjected to mineral carbonation in the presence of NaOH using 2,2′‐bipyridine at a molar ratio of bipy/Fe of 3 : 1. A 10 vol % CO_2_ gas stream was introduced into the reactor at a rate of 0.15 L min^−1^, which was considered the optimum gas flow rate in terms of CO_2_ use/uptake. At a temperature of 60 °C and a pH value between 9.0 and 12.0, the mineral carbonation reaction efficiency was between 40 % and 50 %. Temperatures higher than 60 °C were found to decrease the stability of the iron complex, most likely due to the low solubility of CO_2_ in the aqueous solution at these conditions. In a further investigation, it was reported that by using a relatively high concentration of Na_2_CO_3_ (about 3.2 mol L^−1^) as the carbonate source, up to 50 % precipitation efficiency of iron, as FeCO_3_, can be reached at 80 °C.^[144]^

Wang *et al*. investigated the selective recovery of metals from silicate minerals through metal complexation. Although, their early work focused on mineral carbonation coupled with the conversion of nickel to nickel sulphide by sulphidisation, their more recent studies considered the utilisation of different complexing agents during carbonation. CO_2_ mineralisation and concurrent metal sulphidisation was initially reported as a methodology to recover nickel, iron and manganese from an olivine mineral.^[145]^ The methodology considered the simultaneous introduction of sulphide ions, either in the form of Na_2_S (≤0.6 mol kg^−1^), FeS or H_2_S gas (5 vol %), and CO_2_ at high pressures and temperatures (*P*_CO2_ 10–35 bar, 130–175 °C, 95 vol % when mixed with H_2_S) in an autoclave. Mineral carbonation is a pretreatment for nickel sulphidisation, as it enables the release of Ni^2+^ ions into the aqueous solution from the crystal structure of the olivine matrix. Once in solution, Ni^2+^ can easily be converted into NiS for further treatment by traditional mineral processing techniques (*e.g*., froth flotation) and smelting of sulphide concentrates, while other divalent metal ions, such as Mg^2+^, Ca^2+^ and Fe^2+^, concurrently react with CO_2_ to form stable carbonates. The solubility of nickel sulphide (K_sp_= 8.6×10^−24^) is much lower than that of NiCO_3_ (K_sp_= 9.7×10^−12^). In contrast, MgS (K_sp_= 8.1×10^−4^) and CaS (K_sp_= 1.5×10^−2^) have higher solubility than their corresponding carbonates, i. e. MgCO_3_ (K_sp_= 8.6×10^−6^) and CaCO_3_ (K_sp_= 5.0×10^−9^). Although FeS (K_sp_= 2.5×10^−17^) has a lower solubility than FeCO_3_ (K_sp_= 5.3×10^−9^), the solubility of NiS is even lower than that of FeS. Wang and Dreisinger studied the use of ethylenediaminetetraacetic acid (EDTA) as metal‐complexing ligand for the concurrent recovery of nickel, cobalt, iron, and manganese, from olivine.^[146]^ EDTA aided in removing the passivation layer of carbonates and shifted the kinetic control regime to the dissolution of olivine, particularly at high *P*_CO2_ (20–35 bar) and temperature (135–175 °C). The authors also reported the utilisation of citrate and glycine as complexing reagents, but these reagents did not improve the recovery of nickel from carbon mineralisation.

**Figure 17:**
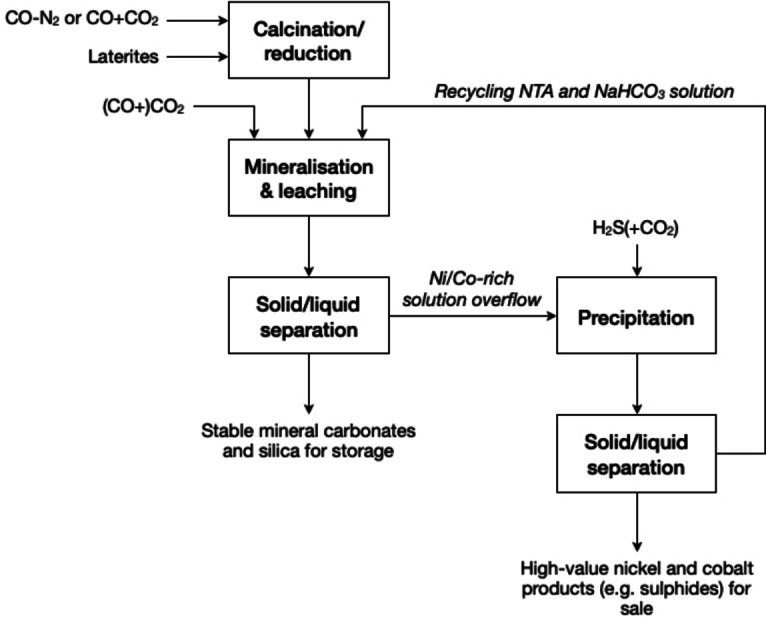
Process flowsheet of the mineral carbonation and selective leaching from laterites (adapted from [149] with permission from Elsevier, copyright 2023).

Nitrilotriacetate sodium salts (NTA) combined with NaHCO_3_ was also investigated by Wang *et al*. as a suitable ligand to achieve simultaneous CO_2_ mineralisation (*P*_CO2_ ≈35 bar and 175 °C, in an autoclave), and selective nickel and cobalt extraction from olivine and nickel‐rich laterite minerals.[^147^, ^148^] Laterite can be highly enriched in serpentine and/or goethite minerals, which are unreactive for CO_2_ mineralisation. Hence, calcination at high temperature (≈700 °C) was carried out before leaching (Figure 17). Leaching was performed with a solution containing NaHCO_3_ (≈1.5 mol L^−1^) and NTA (50–60 mmol L^−1^), and the simultaneous introduction of CO_2_. The solution with nickel and cobalt complexed by NTA was then contacted with H_2_S for the production of high‐value nickel sulphide precipitates, whereas manganese and iron precipitated as carbonates. The solution containing NTA and NaHCO_3_ after metal recovery and residual sulphide removal was recycled back to CO_2_ mineralisation and selective leaching stage.^[149]^ The methodology allowed to recover more than 90 % of nickel and cobalt, and had a 60 % of CO_2_ mineralisation efficiency.

Martins and Martins reported the utilisation of H_2_Na_2_EDTA as complexing reagent for the extraction of tungsten from a scheelite mineral in an aqueous Na_2_CO_3_ solution. The use of H_2_Na_2_EDTA avoids the precipitation of a calcite layer on the surface of the mineral, enhancing the solubilisation of tungsten.^[94]^ In a similar study, Miller *et al*. investigated the use of citrate‐containing solutions for the precipitation of magnesium carbonate nanoparticles.^[150]^ Synthetic forsterite (Mg_2_SiO_4_) was exposed to water‐saturated supercritical CO_2_ that was equilibrated with citrate‐containing solutions of varying concentrations. The complexation of Mg^2+^ ions with citrate anions allowed increasing the proportion of partially hydrated Mg^2+^ in the absorbed water film, and decreased the kinetic barrier for precipitation of anhydrous magnesium carbonate.

## 4 Precipitation Stripping of Metal Carbonates After Solvent Extraction

Enhanced carbonation by solvent extraction (SX) has been proposed as a process intensification methodology by combining the stripping and precipitation unit operations in a single process step – the principle of Maximise Mass, Energy, Time and Space Efficiency in circular hydrometallurgy. Bao *et al*. reported the utilisation of a mixed solvent, composed of water and water‐immiscible tributyl phosphate (TBP), with a definite concentration of acetic acid as leaching solution for the selective dissolution of calcium and magnesium from steelmaking slag. Calcium and magnesium were then converted into carbonates in a subsequent step that comprised crystallisation integrated with SX.^[151]^ TBP was used for the recovery of the acetic acid by SX during the carbonation reaction, which also allows to enhance the crystallisation conversion. After the separation of the carbonate product in the precipitation step, the residual solution – composed of TBP, acetic acid, water and unreacted acetate – was recirculated for reuse in the leaching step.^[152]^ The addition of TBP did not only enhance the separation of acetic acid and the precipitation of (Ca,Mg)CO_3_, but it also prevented its redissolution of the metal carbonate under conditions of high partial CO_2_ pressures or high stirring speeds.

**Figure 18:**
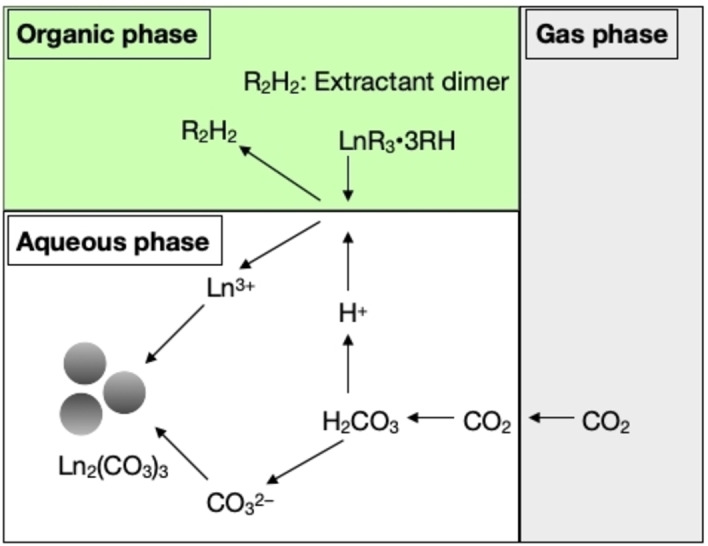
Conceptual diagram for precipitation stripping of the REE‐loaded solvent extractant using aqueous CO_2_. (adapted from [154] with permission from The American Chemical Society, copyright 2001).

In a traditional‐commercial SX process, metals are typically stripped from loaded organic phase by acidic aqueous solutions if acidic extractants are used for metal extraction. Metals can be precipitated from the acidic stripping solution as insoluble hydroxides, oxalates or carbonates, by the neutralisation with a base, addition of oxalic acid or addition of soda ash, respectively.^[153]^ This process, however, can be simplified by emulsifying the metal‐loaded solvent extractant with aqueous CO_2_ for the direct precipitation of metals ions from the loaded organic phase, i. e. the combination of stripping and precipitation. This can be considered as a form of process intensification. Precipitation stripping of REE carbonate powders from REE‐loaded organic carboxylate solutions was demonstrated by Konishi and Noda under autoclave conditions at *P*_CO2_ up to 30 bar and temperatures between 10 and 80 °C.^[154]^ A commercial tertiary aliphatic monocarboxylic acid (Versatic Acid 10) diluted with Exxsol D80 (an aliphatic hydrocarbon) was used as the solvent for extraction. During the extraction operation, the aqueous solution pH was increased to about 6.0 by addition of a dilute NaOH solution. A conceptual diagram for the precipitating stripping mechanism of the REE‐loaded solvent is depicted in Figure 18. The dissolution of CO_2_ in water entails the dissociation of the H_2_CO_3_ in protons and carbonate ions (Equations (1)–(4)). The released H^+^ and CO_3_^2−^ ions act as stripping (Equation (27)) and precipitating (Equation (28)) agents, respectively. The discharge of the trivalent lanthanide (REE) ions, Ln^3+^, from the organic phase shifts the equilibrium of the stripping process (Equation (29)) towards the regeneration of the extractant dimer (R_2_H_2_). The precipitation stripping mechanism is favoured when the partial CO_2_ pressure *P*_CO2_ is increased, whereas the temperature had a major effect on particle size distribution of formed carbonates.

(27) $$Stripping:\;2LnR_{3}\cdot 3RH_{\left(org\right)}+6H_{\left(aq\right)}^{+}\to 2{Ln}_{\left(aq\right)}^{3+}+6R_{2}H_{2\left(org\right)}$$

(28) $$Precipitation:\;2{Ln}_{\left(aq\right)}^{3+}+3\;\;CO_{3\left(aq\right)}^{2-}\to {Ln}_{2}{\left(CO_{3}\right)}_{3\left(s\right)}$$

(29) $$Overall\;reaction:3CO_{2\left(g\right)}+3H_{2}O_{\left(aq\right)}+2LnR_{3}\cdot 3RH_{\left(org\right)}\to {Ln}_{2}{\left(CO_{3}\right)}_{3\left(s\right)}+6R_{2}H_{2\left(org\right)}$$

## 5 Coupled Reaction and Solvent Extraction Process

Solvent extraction represents an effective methodology to lower the acidity of the aqueous solution by transferring the acid into the organic phase, to be recovered afterward ideally by just adding water – principle of Regenerate Reagents. Different solvents have been tested for acid extraction from aqueous solutions.[^155^, ^156^, ^157^, ^158^] This approach can be coupled with mineral carbonation, so that the precipitation of carbonate compounds is promoted in the acid‐free aqueous phase – the principle of Maximise Mass, Energy, Time and Space Efficiency. Zhou *et al*. studied the synthesis of Li_2_CO_3_ from LiCl by combining the carbonation reaction with solvent extraction.^[159]^ They called it a coupled reaction and solvent extraction process. In aqueous medium, the reaction of LiCl and CO_2_ is not spontaneous at ambient conditions due to the formation of HCl in the aqueous phase. Hydrochloric acid is removed from the aqueous phase by solvent extraction using the tertiary amine tri‐*n*‐octylamine (N235) as the extractant and *iso*‐octanol as (active) diluent. The overall reaction for the carbonation and solvent extraction process is given in Equation (30), where the organic phase is represented by the letter *S* and the overbar means species in the organic phase. According to the authors, experiments conducted in an open reactor at room temperature showed that the product sample was pure Li_2_CO_3_ with a bimodal particle‐size distribution and a larger number of small crystals. It was observed that with short reaction times, large organic‐to‐aqueous ratio (O/A=3), and surfactant addition (Triton X‐100), it is possible to increase the volume of small particle crystals.

(30) $$2{Li}_{\left(aq\right)}^{+}+2{Cl}_{\left(aq\right)}^{-}+CO_{2\left(g\right)}+H_{2}O_{\left(aq\right)}+2{\bar{S}}_{\left(org\right)}\vec{\leftarrow }{Li}_{2}CO_{3\left(s\right)}+2{\bar{\left(S\cdot HCl\right)}}_{\left(org\right)}$$

**Figure 19:**
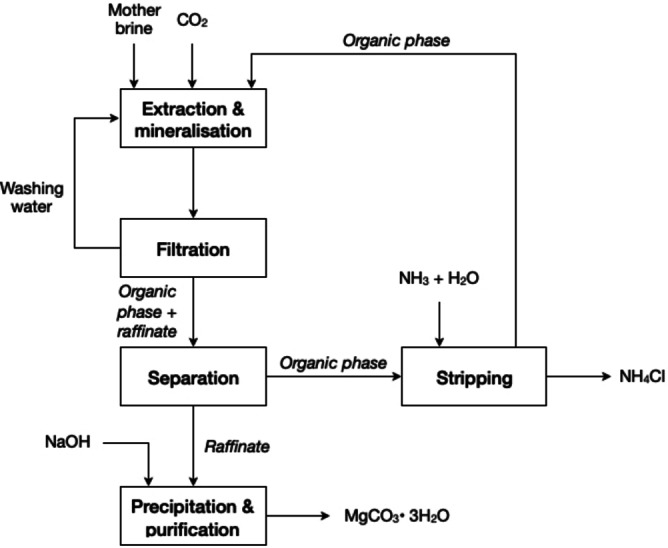
Conceptual diagram for acid extraction combined with CO_2_ mineralisation (ECM) process for lithium enrichment from mother brine (adapted from [160] with permission from The American Chemical Society, copyright 2017).

A similar approach was followed by Chen *et al*. who studied the extraction of HCl with different amines to concurrently convert MgCl_2_ into MgCO_3_ from a Mg/Li‐rich brine. Similarly, the corresponding reaction mechanism is described by Equation (31).^[160]^ The equilibrium concentration of Mg^2+^ ions depends upon the pH of the aqueous phase, which is co‐dependent on the basicity of the organic amine and, consequently, defined by its substituent groups that influence the inductive and steric hindrance effects. Increasing the number of substituent groups and the alkyl chain length increases the electron density on the nitrogen atom, thereby increasing the potential to attract protons (inductive effect), but also increases the resistance to react with the protons (steric hindrance effect). The longer the substituent on the tertiary amine, the lower the solubility in water and the lower the vapor pressure. The effect of different amines and diluents, as well some other operational variables, were reported. Figure 19 describes the conceptual diagram for acid extraction combined with CO_2_ mineralisation for lithium enrichment from mother brine. Initially, the mother brine was contacted with CO_2_ and the amine‐based solvent extractant. The dissolution of magnesium and its conversion into carbonate is determined by the extent at which H_2_CO_3_ forms and decomposes in aqueous solution (H_2_CO_3_/HCO_3_^−^ at pK_a1_= 6.35 and HCO_3_^−^/CO_3_^2−^at pK_a2_= 10.2, at 25 °C). A blank experiment confirmed that under optimal conditions, about 99 % Li and 67 % Mg could be recovered by bubbling CO_2_ at a rate of 60 mL min^−1^ during 120 min. Trioctyl amine (TOA), at 50 vol % was used as extractant, at an organic‐to‐aqueous phase ratio (O/A) of 8. Sodium hydroxide was used to precipitate magnesium from aqueous phases with relatively a low Mg/Li ratio, *i.e*. 20 : 6, but less lithium was recovered (84 %). Wash water was separated from the organic phase by filtration, whereas the organic phase was separated from the raffinate by decantation. The HCl extracted by the amine could not be stripped by water, hence, an aqueous ammonia solution was used instead. The stripping reaction converted R_3_NH^+^Cl^−^ into NR_3_, while NH_4_Cl was separated by stripping the aqueous phase.

(31) $${MgCl}_{2\left(aq\right)}+\;2{\bar{NR_{3}}}_{\left(org\right)}+CO_{2\left(g\right)}+H_{2}O_{\left(aq\right)}\vec{\leftarrow }2{\bar{R_{3}NH^{+}\cdot {Cl}^{-}}}_{\left(org\right)}+MgCO_{3\left(s\right)}$$

**Figure 20:**
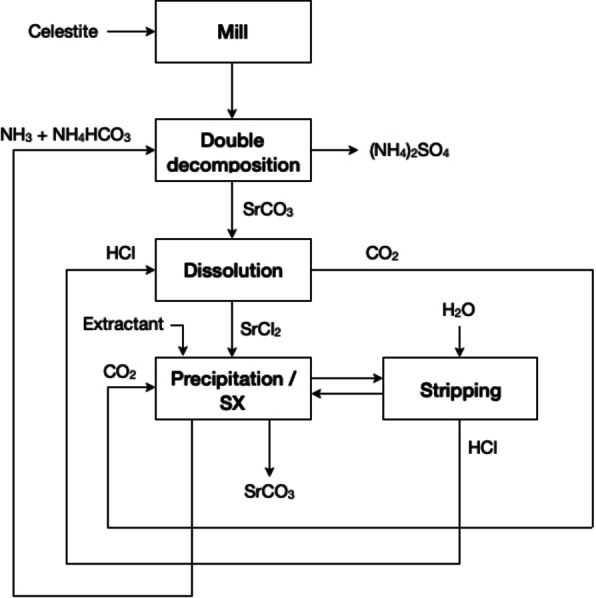
Conceptual diagram for double decomposition process coupled with solvent extraction of acid and precipitation of strontium carbonate (adapted from [161] with permission from Elsevier, copyright 2005).

Xu *et al*. studied the reaction between CO_2_ and SrCl_2_ coupled with solvent extraction of the HCl formed in the reaction.^[161]^ The method was developed as an alternative to the high‐temperature (1000–1200 °C) conversion process of the celestite mineral (SrSO_4_) into strontium sulphide (SrS) and/or polysulphides. The process entails the double decomposition of the sulphate mineral in the presence of mixed solution composed of NH_3_ and NH_4_HCO_3_ at 90 °C (Figure 20). Once in solution, strontium precipitates as part of a mixed carbonate compound. The formed carbonate is a mixed intergrowth with gangue minerals, which is further processed by dissolution with HCl. The tertiary amine N235 diluted with *iso*‐amyl alcohol, was used for the extraction of the acid. With the concurrent introduction of CO_2_ into the system, more than 90 % of the strontium was converted into carbonate when the extractant concentration was above 0.9 mol L^−1^. According to the authors, a high partial CO_2_ pressure did not result in a significant difference in terms of acid extraction yields, relative to atmospheric condition experiments, but the reaction was faster at high CO_2_ pressures.

Zhang *et al*. reported the use of aqueous CO_2_ for stripping lithium from the organic phase composed by the extractants LIX 54 (a mixtures of β‐diketones) and trioctylphosphine oxide (TOPO).^[137]^ The highest extraction yield of lithium was achieved by the combined effect of both extractants at a ratio of 1 : 1 and at a pH about 11.5. Lithium stripping was enhanced by increasing the concentration of carbonate ions in the reaction system, as this leads to reducing the solution′s pH to about 7.0. A lithium yield of about 98 % was obtained at a pH of 7.5, which was comparable to the stripping efficiency obtained with HCl.

## 6 Other Applications

**Figure 21:**
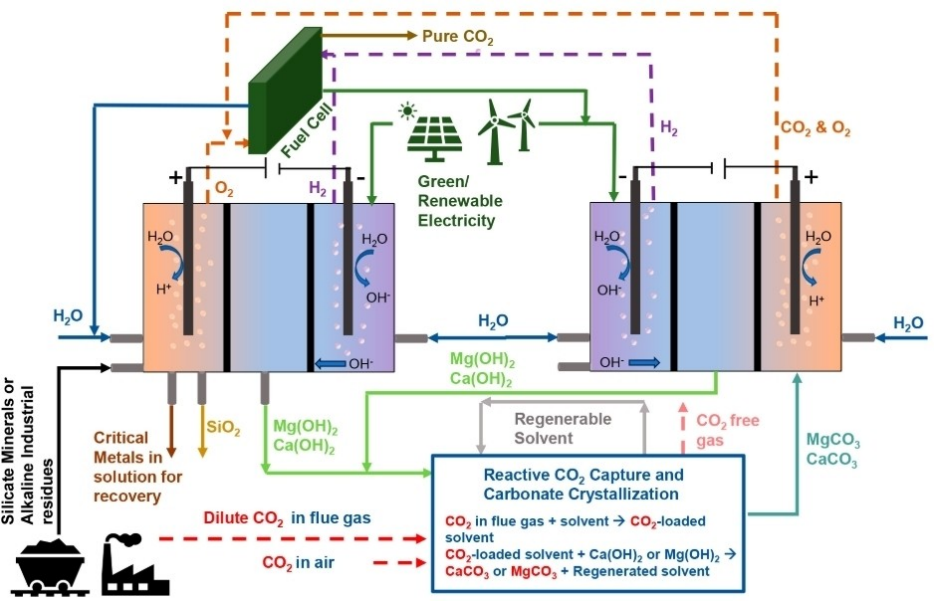
Process diagram of the integrated approach for integrating CO_2_ capture, conversion, and storage via mineralisation. Adapted from reference [162] with permission from The American Chemical Society, copyright 2024.

Ochonma *et al*. performed feasibilities studies on reactive crystallisation for integrating CO_2_ capture, conversion, and storage via mineralisation (see Figure 21).^[162]^ The methodology considers the utilisation of naturally occurring silicates minerals and/or alkaline industrial residues as input materials. These are subjected to an electrochemical desilication process for producing calcium and magnesium hydroxide, hydrogen and oxygen gas, silica, and a leachate comprising other metals normally present in the input materials (*e.g*., manganese, titanium, iron). The produced Ca/Mg hydroxides are then contacted with CO_2_‐loaded solvents, such as sodium glycinate and ethanolamine, to produce Ca/Mg carbonates while concurrently regenerating the CO_2_ absorbing solvent for its reuse. These carbonates can be used for permanent or temporary storage of CO_2_. For applications that may require high‐purity CO_2_, Ca/Mg‐carbonates can be electrochemically converted to Ca/Mg‐hydroxide while concurrently producing H_2_ and O_2_ to be used in fuel cells, for instance; thus, complying with the principle of Electrify Processes Wherever Possible. By coupling both capture and mineralisation in a single step can significantly save costs on capital expenditures (CAPEX).

**Figure 22:**
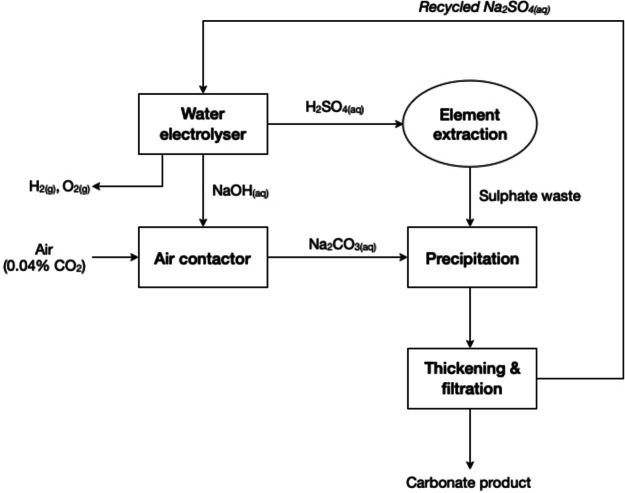
Conceptual diagram for carbon mineralisation and H_2_SO_4_ production (adapted from [163] with permission from The American Chemical Society, copyright 2023).

Lammers *et al*. reported the proof‐of‐concept of an integrated process for CO_2_ mineralisation with electrolytic H_2_SO_4_ production.^[163]^ The proposed methodology requires the input of sulphate raw materials with sufficient acid neutralising potential, such as rock phosphorus or mine tailings. According to the process described in Figure 22, air containing 0.04 % CO_2_ is introduced directly into the precipitation reactor filled with a slurry of ground solid gypsum. In the precipitation reactor, CO_2_ reacts with Ca(OH)_2_ produced in the water electrolyser to form CaCO_3_ precipitates. The total energy requirement of the process is about 15 GJ ton^−1^ CO_2_ sequestered, considering an energy intensity of acid production of 0.72 MJ mol^−1^ H_2_SO_4_, where the energy can be recovered by hydrogen combustion in a fuel cell with 60 % efficiency. Compared with the energy demand of current technologies for direct air capture and carbon sequestration (11 GJ ton^−1^ CO_2_), this methodology could require less than 7 GJ per tonne of CO_2_ sequestered under further optimisations. Based on preliminary economic calculations, the total cost for producing 1 tonne of acid by this method was estimated to be 112 USD. Similar to the process reported by Ochonma *et al*., this process also exemplifies the principle of electrification using CO_2_ as benign chemical for the production of H_2_SO_4_.

Other chemical reagents, such as acetic, citric and oxalic acids, can also be produced from CO_2_. For instance, citric acid can be produced through the photosynthetic conversion of CO_2_ by using micro‐organisms,^[164]^ whereas acetic acid can be produced by mixing CO_2_ with syngas (a mixture of CO and H_2_ gas) or with methanol and hydrogen gas.[^165^, ^166^] Sustainable processing routes for the production of oxalic acid have also been investigated, which include the utilisation of CO_2_ combined with biomass waste streams.^[167]^

Another interesting approach in hydrometallurgy is the utilisation of diethyl carbonate (DEC) as an extractant for the recovery of gold from copper‐rich sources by solvent extraction from chloride solutions.^[168]^ Diethyl carbonate is characterised by its low toxicity and high biodegradability, and it can be produced by condensation of ethanol and CO_2_ under increased temperature and pressure. The production of DEC is very sustainable if bioethanol can be used instead of ethanol prepared via the conventional chemical processes.

Recent studies have shown that mixtures of CO_2_+O_2_ perform excellently for in‐situ leaching (ISL) of uranium from sandstone ores.^[169]^ Here, O_2_ acts as the oxidising agent to convert all the uranium present in the less soluble tetravalent state in the more soluble hexavalent state (formation of uranyl ions), whereas CO_2_ acts as a complexing agent to form the anionic tetracarbonato uranyl complex. The chemistry behind the *in‐situ* leaching is complex, with many coupled reactions involved.^[170]^ For instance, the protons formed upon reaction of CO_2_ and water are consumed for the dissolution of UO_2_ and this drives the CO_2_/H_2_O equilibrium to the right, to hydrogencarbonate formation. Meanwhile the HCO_3_^−^ ions together with O_2_ can oxidise UO_2_ directly to the tetracarbonato uranyl complex. The CO_2_+O_2_ *in‐situ* leaching approach was tested at different locations in P.R. China, but is was also demonstrated in the Zinda Pir Anticline (ELZPA) ore deposit in Pakistan.^[171]^ Phytomining (agromining) of nickel or other metals with hyperaccumulator plants is an example of low‐impact mining, since it permits a huge preconcentration of the metal without human interference, and at low cost.^[172]^ Since the plants used for phytomining are taking up CO_2_ from the air while growing, phytomining is in fact a CO_2_‐negative process (although part of the CO_2_ is released again when the biomass is burnt to recover the nickel from the ashes). When nickel hyperaccumulator plants are grown on soils rich in olivine or serpentine, the plant roots assist in the weathering of the soils and this activates the soils towards mineral carbonation.^[173]^

## 7 Carbon Dioxide and the Twelve Principles of Circular Hydrometallurgy

If one assesses how the research activities reviewed in the previous sections are addressing the twelve principles of circular hydrometallurgy, it is evident that most of the research done so far is related to the Prevent Waste principle. Indeed, CO_2_ is considered as a waste that needs to be safely disposed of. This can be done by converting the CO_2_ gas into inert materials by mineral carbonation. When these carbonated minerals can find application in construction materials, it is a nice example of turning waste into valuable materials. Mineral carbonation is an excellent approach to reduce Scope 1 emissions, i. e. GHG emissions that are produced or controlled by the metallurgical plant. The use of CO_2_ for neutralisation of alkalinity, or the use of CO_2_‐derived chemicals such as Na_2_CO_3_, NaHCO_3_ and (NH_4_)_2_CO_3_, as leaching reagents are examples of the principle Use Benign Chemicals, but also the principle Reduce Chemical Diversity, since these are all well‐known, easily available chemicals.

The precipitation stripping of metals from the loaded organic phase by solvent extraction is a representative example of the principle Maximize Mass, Energy, Space, and Time Efficiency, because precipitation stripping combines two unit operations, i. e. stripping and precipitation, in a single step. Hence, precipitation stripping is a form of process intensification. Also the coupled reaction and solvent extraction process to precipitate Li_2_CO_3_ in an aqueous LiCl solution by CO_2_ with the simultaneous transfer of the HCl formed to an immiscible organic phase, is an example of process intensification. If the HCl can be recovered from the organic phase by water instead of using a base for neutralisation and, if the stripped HCl in the aqueous phase can be further concentrated to useful concentrations, this process is an example of the principle Regenerate Reagents. However, the potential of CO_2_ for regenerating reagents is still largely unexplored. The coupled reaction and solvent extraction process mentioned above could be used to precipitate different types of carbonates, with regeneration of the acids used for leaching in the hydrometallurgical process. Indeed, the reaction of CO_2_ and water gives unique opportunities to re‐introduce protons in the system and hence regenerating acids, but the process must be coupled to solvent extraction to remove the acids from the aqueous equilibrium to shift the equilibrium to the right. One of the challenges is to find suitable solvents with high enough affinity for the acid, so that the acid can be efficiently extracted from the aqueous phase, but the affinity should not be too high because, otherwise, it will be impossible to strip the acid from the loaded organic phase by (hot) water. Stripping with a base is not an option, because this will neutralise the acid and will form a salt.

The *in situ* leaching of uranium ores with CO_2_+O_2_ is obviously an example of the principle Combine Circular Hydrometallurgy with Zero‐Waste Mining. The principle Electrify Processes Wherever Possible is illustrated in the electrochemical desilication process, where SiO_2_ recovery is combined with CO_2_ capture, conversion, and storage via mineralisation.^[162]^ Given the strong research interest in the electrochemical transformation of CO_2_ in useful chemicals, it is to be expected that there are still a lot of opportunities for electrochemical synthesis of useful reagents for hydrometallurgy, using CO_2_ as a renewable feedstock. To give one example, the electrochemical and photochemical synthesis of formic acid from CO_2_ can be mentioned.[^174^, ^175^, ^176^] Formic acid finds use as a mild lixiviant or a reducing agent in hydrometallurgy.[^177^, ^178^, ^179^]

The other principles of circular hydrometallurgy not mentioned so far have not been explicitly addresses in this review paper, although we are convinced that there are opportunities. For instance, online monitoring of CO_2_ concentrations by sensors could assist in process control of hydrometallurgical processes involving CO_2_ (Implement Real‐Time Analysis and Digital Process Control), and CO_2_ could help in the safe disposal of toxic and harmful elements (Safely Dispose of Potentially Harmful Elements).

## 8 Conclusions and Future Perspectives

Carbon dioxide finds application in (circular) hydrometallurgy for the neutralisation of alkaline solutions and solids, as main constituent of different reagents such as Na_2_CO_3_, NaHCO_3_ and (NH_4_)_2_CO_3_, and for more novel applications such the coupled reaction and solvent extraction process to precipitate metal carbonates and regeneration of acids. However, by far most applications have focused on the use of (waste) CO_2_ for mineral carbonation.

Mineral carbonation, however, suffers from high economic costs and slow reaction rates due to the cost of grinding and heat pre‐treatment of precursor materials. Furthermore, during indirect mineral carbonation, a significant volume of solid material remains unreactive after leaching – possible containing a significant concentration of valuable metals – that will require further processing to be safely disposed. This implies that the whole sequestration process may result in more CO_2_ emissions than the sequestered CO_2_ due to the requirement of high energy inputs and the use no‐so‐easy‐to‐regenerate lixiviants. A successful carbonation process must manage to precipitate valuable carbonate products and recycle most of the additional chemicals used.

Despite the growing interest in carbon capture and utilisation, there is still potential to make mineral carbonation a profitable process by considering waste CO_2_ as a valuable reagent within hydrometallurgical flowsheets. For instance, since large volumes of CO_2_ are released during the leaching of carbonate minerals (both ore and gangue minerals) with acids, this can be avoided by considering alternative leaching reagents that can consume rather than emit CO_2_. Thus, CO_2_ emitted during leaching can be captured and be reused for the precipitation of divalent ions in the form of metal carbonates MCO_3_, with M representing calcium(II), magnesium(II), manganese(II) or iron(II). These carbonates are environmentally friendly and are similar to the natural carbonate minerals incorporating these ions, i. e. calcite (or aragonite), magnesite, rhodochrosite and siderite, respectively. The carbonates can be precipitated by addition of a carbonate salt, e. g., ammonium (hydrogen)carbonate or sodium (hydrogen)carbonate. This, however, may entail the consumption of significant amounts of chemicals, and it does not reintroduce protons to the system for the regeneration of the acid. Recent studies have considered the integration of mineral carbonation with solvent extraction for the concurrent regeneration of the acid from the aqueous solution and the precipitation of metal carbonates. Likewise, carbonation coupled with metal complexation agents can enhance the reactivity and solubility of metal ions released during mineral carbonation reactions, as the kinetic of the carbonation reaction can be further accelerated.

CO_2_ can be used in hydrometallurgy either directly or indirectly as a reactant to enhance metal leaching and precipitation processes. It forms carbonate species, increasing the solubility of metal ions from ores and facilitating their recovery. Additionally, CO_2_ can promote the precipitation of metal carbonates, aiding in metal purification and separation from impurities. CO_2_ can also be used for the generation of chemical reagents *in‐situ* by, for example, combining it with an aqueous ammonia solution for the preparation of (NH_4_)_2_CO_3_. This environmentally benign approach reduces the need for harsh chemicals and energy‐intensive processes, making it a sustainable option for metal extraction and processing.

In conclusion, rather than considering CO_2_ as a waste product, it must be understood that CO_2_ can also be a valuable reagent in hydrometallurgy. There is still a lot of untapped potential for the use of CO_2_ in this domain. The twelve principles of circular hydrometallurgy can be a guide for the further exploration of applications of CO_2_ in hydrometallurgy. Probably the most valuable applications will be as a renewable feedstock for the synthesis of chemicals to be used as reagents in hydrometallurgy, for neutralisation of excess base after alkaline leaching, for precipitation stripping of metal carbonates from the loaded organic phase after solvent extraction, and for the coupled reaction of precipitation of carbonates and the regeneration of acids by solvent extraction.

## Conflict of Interests

There are no conflicts of interest to declare.

## Biographical Information

*Dr. Rodolfo Marin Rivera′s background focuses on process engineering, metallurgy, and sustainable resource recovery. He graduated with a master degree in Metallurgical Engineering from RWTH Aachen (Germany) and PhD in Chemical Engineering from KU Leuven (Belgium). Dr. Rivera′s research has focused on the utilisation of both aqueous and non‐aqueous solvents for selective metal recovery from a diversity of raw materials, including electronic waste, industrial solid waste residues, aerospace materials and meteorites. He is currently working on the development of innovative process technologies that are both energy and resource efficient, consuming minimal amounts of reagents and generating minimal waste*.

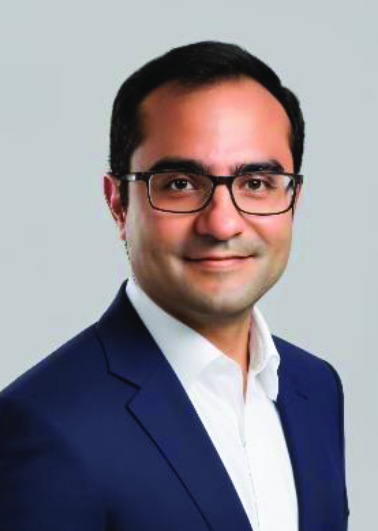

## Biographical Information

*Prof. Koen Binnemans is an inorganic chemist who is combining fundamental and applied research in the fields of metallurgical chemistry and hydrometallurgy. He is head of the SOLVOMET group at the Department of Chemistry of KU Leuven (Belgium). He has published more than 600 papers in international journals. His work has been cited 37 500 times (h‐index=90) according to Web of Science. He has been awarded two ERC Advanced Grants (SOLCRIMET and CIRMET). He has developed the 12 Principles of Circular Hydrometallurgy*.